# How to Separate Kinase Inhibition from Undesired Monoamine Oxidase A Inhibition—The Development of the DYRK1A Inhibitor AnnH75 from the Alkaloid Harmine

**DOI:** 10.3390/molecules25245962

**Published:** 2020-12-16

**Authors:** Anne Wurzlbauer, Katharina Rüben, Ece Gürdal, Apirat Chaikuad, Stefan Knapp, Wolfgang Sippl, Walter Becker, Franz Bracher

**Affiliations:** 1 Department of Pharmacy—Center for Drug Research, Ludwig-Maximilians University, 81377 Munich, Germany; Dr.Anne.Wurzlbauer@t-online.de; 2 Institute of Pharmacology and Toxicology, RWTH Aachen University, 52074 Aachen, Germany; katharina@rueben.biz (K.R.); wbecker@ukaachen.de (W.B.); 3 Institute of Pharmacy, Martin Luther University Halle-Wittenberg, 06120 Halle, Germany; eceguerdal@gmail.com (E.G.); wolfgang.sippl@pharmazie.uni-halle.de (W.S.); 4 Institute of Pharmaceutical Chemistry and Structural Genomics Consortium, Buchmann Institute for Molecular Life Sciences (BMLS), Goethe-University Frankfurt, 60438 Frankfurt, Germany; chaikuad@pharmchem.uni-frankfurt.de (A.C.); knapp@pharmchem.uni-frankfurt.de (S.K.)

**Keywords:** alkaloid, harmine, DYRK1A, monoamine oxidase A, docking studies, co-crystallization, structure–activity relationships

## Abstract

The β-carboline alkaloid harmine is a potent DYRK1A inhibitor, but suffers from undesired potent inhibition of MAO-A, which strongly limits its application. We synthesized more than 60 analogues of harmine, either by direct modification of the alkaloid or by de novo synthesis of β-carboline and related scaffolds aimed at learning about structure–activity relationships for inhibition of both DYRK1A and MAO-A, with the ultimate goal of separating desired DYRK1A inhibition from undesired MAO-A inhibition. Based on evidence from published crystal structures of harmine bound to each of these enzymes, we performed systematic structure modifications of harmine yielding DYRK1A-selective inhibitors characterized by small polar substituents at N-9 (which preserve DYRK1A inhibition and eliminate MAO-A inhibition) and beneficial residues at C-1 (methyl or chlorine). The top compound **AnnH75** remains a potent DYRK1A inhibitor, and it is devoid of MAO-A inhibition. Its binding mode to DYRK1A was elucidated by crystal structure analysis, and docking experiments provided additional insights for this attractive series of DYRK1A and MAO-A inhibitors.

## 1. Introduction

Protein phosphorylation is a major regulatory mechanism of nearly every cellular process, and many human diseases involve aberrant protein kinase activities. Specific kinase inhibitors serve as valuable research tools to elucidate the individual roles of the more than 500 human protein kinases in physiological and pathological processes. DYRK1A (dual-specificity tyrosine phosphorylation-regulated kinase 1A) is a protein kinase that has important functions in neuronal development and cell cycle control, and attracts increasing attention as a possible drug target [1,2]. The *DYRK1A* gene is localized on human chromosome 21, and substantial evidence supports the hypothesis that DYRK1A overexpression significantly contributes to the altered brain development and intellectual disability in individuals with Down syndrome [3]. Furthermore, DYRK1A has been implicated in neurodegenerative processes [4], certain cancers [5] and regulation of pancreatic β-cell proliferation, suggesting inhibition of DYRK1A as a novel therapeutic strategy in diabetes [6,7]. Thus, inhibitors of DYRK1A are of interest not only as chemical probes for the functional characterization of DYRK1A’s role in these pathological processes but also as potential therapeutics.

Meanwhile, numerous small molecules from different chemotypes have been identified inhibiting DYRK1A, as recently reviewed by Jarhad et al. [2]. Since then, additional chemotypes have been identified as DYRK1A inhibitors [8,9,10,11]. Among them, the β-carboline alkaloid harmine was originally identified in 2007 as a DYRK1A inhibitor [12], and it was later confirmed to be selective for DYRK1A in kinome profiling screens [13]. Harmine inhibits DYRK1A and the closely related isoform DYRK1B in an ATP competitive manner with similar IC_50_ values of about 33–170 nM, while DYRK2 and DYRK3 are only inhibited at much higher concentrations, and DYRK4 is basically resistant to harmine [14]. Importantly, harmine is a lipophilic, cell permeant compound, and it inhibits DYRK1A in cell based assays at similar concentrations as in biochemical assays [14]. Therefore, harmine is frequently used to corroborate the presumed role of DYRK1A in signaling events.

Unfortunately, the use of harmine as a DYRK1A inhibitor is hampered by the fact that it inhibits the enzyme monoamine oxidase A (MAO-A) at even lower concentrations than DYRK1A (IC_50_ = 5 nM [15]). This cross-reactivity limits the use of harmine as a DYRK1A inhibitor in vivo and precludes potential clinical application. Nevertheless, harmine is a valuable lead structure to develop an optimized β-carboline as a selective DYRK1A inhibitor with no effect on MAO-A.

**Figure 1:**
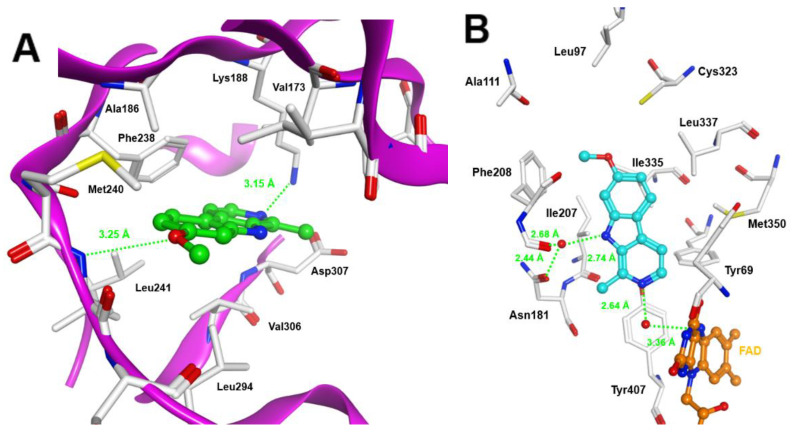
(**A**) Binding modes of harmine (carbon atoms colored in green) in DYRK1A (PDB 3ANR), and (**B**) harmine (carbon atoms colored light blue) in MAO-A (PDB 2ZGX). Hydrogen bonds are indicated by dashed green lines, water molecules as red balls. Distances are given in Å.

Based on the co-crystal structures of harmine bound to DYRK1A ([16]; PDB 3ANR) and to MAO-A ([17]; PDB 2ZGX), we hypothesized that the β-carboline scaffold could be modified to obtain a DYRK1A inhibitor devoid of MAO-inhibiting activity. These co-crystal structures indicate that in DYRK1A the methoxy group of harmine forms a hydrogen bond with the backbone Leu241 in the hinge region, and is fully occupying the available space in that region with this methoxy group. The nitrogen atom in the pyridine ring A forms a hydrogen bond with the side chain of Lys188. The central pyrrole NH is oriented towards an open outer pocket, and does not participate in noteworthy interactions with the protein. On the other hand, binding in MAO-A takes place through a network of hydrogen bonds with water molecules, formed by both the pyridine nitrogen atom and the pyrrole NH in ring B. The methoxy group does not contribute significantly to binding, and only partly fills the available space in the pocket (Figure 1).

Reniers et al. [18] already demonstrated that the MOA-A inhibitory activity of harmine can even be enhanced by replacing the methoxy group at ring C by lipophilic ethers (cyclohexyl, benzyl), whereas a polar (*N*,*N*-dimethylamino)ethoxy residue (see compound **AnnH62** of this investigation) reduced MAO-A inhibition by a factor of 60. On the other hand, the co-crystal structure suggested that substituents at the central pyrrole nitrogen, which prevent formation of the hydrogen bond network, might lead to reduced MAO-A inhibition. This evidence was confirmed recently by Haider et al. with harmine-based 7-triazolylmethyl ethers [19]. In contrast, the methoxy group appeared essential for DYRK1A inhibition, while larger ether residues at C-7 should be detrimental due to steric hindrance in the pocket. Substitution at N-9 should be tolerated here from a steric point of view, and Drung et al. demonstrated a positive effect of an *n*-heptyl residue at N-9 for DYRK1A inhibition, combined with a significant, but not complete, reduction of MAO-A inhibition [20].

Based on the evidence available at the beginning of our project, structure modifications at multiple sites of harmine appeared worth investigating. Therefore, we set up a comprehensive synthesis program, which ultimately led to the development of harmine analogue **AnnH75** as a potent DYRK1A inhibitor devoid of any MAO-A inhibitory potency. The central findings of this investigation were confirmed later by Balint et al. [21], who investigated a closely related, but significantly smaller, set of harmine analogues. The comprehensive pharmacological characterization of **AnnH75** has already been published by our laboratory [22]. In 2018, Kumar et al. performed a number of variations at C-1 and C-3 of harmine, but this work was exclusively aimed at improving selectivity over other protein kinases, and MAO-A inhibition was not investigated [23]. In this paper, we describe the complete medicinal chemistry aspects of this project, including the enzyme kinetic characterization that provided new insight into structure–activity relationships, and led to the selection of **AnnH75** as the most promising DYRK1A inhibitor for further pharmacological characterization.

## 2. Results

**Figure 2:**
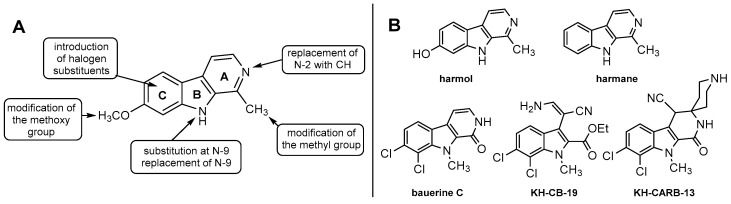
(**A**) Intended modifications of harmine. (**B**) Related alkaloids harmol and harmane, and kinase inhibitors with the uncommon dichloroindole/dichloro-β-carboline motif: bauerine C and synthetic kinase inhibitors derived thereof.

Harmine, as well as the related alkaloids harmol and harmane were, inspired by the investigations of Frost et al. [24], included into our characterization. In the course of a systematic modification of harmine, easy-to-perform direct functionalizations of the native alkaloid were performed in a first series of synthesized analogues, typically by ring halogenation reactions, modification of the 1-methyl and 7-methoxy group, and alkylations or acylation at N-9. In a second series, analogues of harmine were prepared de novo, in order to achieve introduction of hitherto not accessible substituents at rings A and C, and preparation of 2- and 9-desaza analogues (Figure 2A). Of special interest here was the generation of 7,8-dichlorinated β-carbolines, since our previous investigations on analogues of the dichlorinated 1-oxo-β-carboline alkaloid bauerine C provided potent and selective kinase (CLK1, DYRK1A, PIM1) inhibitors with a very unusual binding mode; namely, formation of a halogen bond with the hinge region in the active site [25,26,27] (Figure 2B).

### 2.1. Chemistry

#### 2.1.1. Direct Functionalization of Harmine

##### Direct Modifications at Ring A

**Scheme 1:**
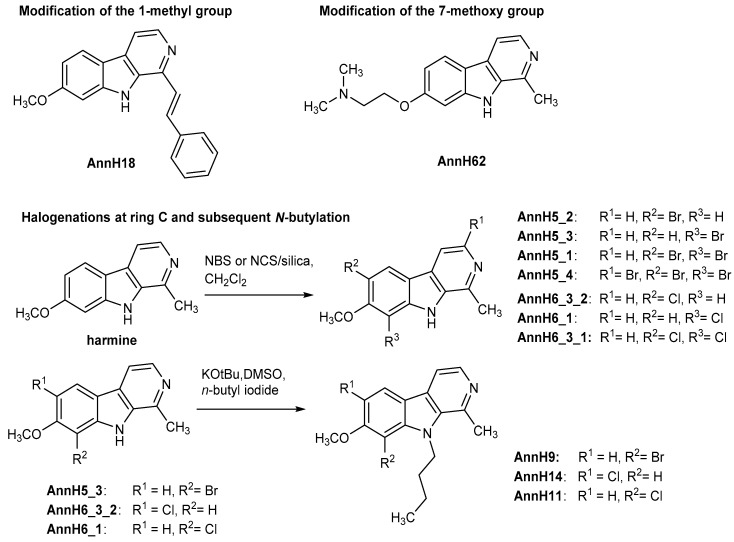
Compounds obtained by modification of substitution patterns in rings A and C of harmine.

Condensation of harmine at the CH-acidic 1-methyl group with benzaldehyde gave the known styryl derivative **AnnH18** [28] (Scheme 1).

##### Direct Modifications at Ring C

Due to the interesting binding mode of the above mentioned dichlorinated kinase inhibitors from our previous work (Figure 2B), we investigated the influence of additional halogen substituents on ring C of harmine. Following published methods [20,29,30], halogenation of harmine with *N*-chlorosuccinimide (NCS) and *N*-bromosuccinimide (NBS) in dichloromethane in the presence of silica gave 6- and 8-monosubstituted products (**AnnH6_1**, **AnnH6_3_2**, **AnnH5_2**, **AnnH5_3**) along with 6,8-dihalogenated harmines (**AnnH6_3_1**, **AnnH5_1**). Using an excess of NBS gave 3,6,8-tribromoharmine **AnnH5_4** in poor yield (4%).

As alkylation of N-9 promised reduction of MAO-A-inhibitory activity, we converted the three readily available monohalogenated compounds (**AnnH5_3**, **AnnH6_1**, **AnnH6_3_2**) into their *N*-butyl derivatives **AnnH9**, **AnnH11** and **AnnH14** by N-deprotonation and subsequent reaction with *n*-butyl iodide (Scheme 1).

Further, we prepared aminoalkyl ether **AnnH62**, following the protocol of Reniers et al. [18] by *O*-alkylation of harmol. The authors found reduced MAO-A-inhibitory activity for this compound compared to harmine, but they did not investigate the influence of this modification on DYRK1A inhibitory activity (Scheme 1).

##### Modifications at Ring B—Acetylation and Alkylation at N-9

The central pyrrole ring, especially the NH function, was of special interest, since the latter contributes significantly to binding of harmine to MAO-A via a hydrogen bond to a surrounding water molecule, but is obviously not involved in binding of the alkaloid to DYRK1A. Consequently, substitution at N-9 was expected to have a major impact on the selectivity profile of harmine derivatives. This prompted us to synthesize a large number of derivatives bearing residues at N-9 covering a broad spectrum of sizes and polarities.

*N^9^*-Acetylharmine (**AnnH59**) was obtained accidentally by treating harmine with piperonal and acetic anhydride, following a method of Cao et al. [31], aimed at the synthesis of a (methylenedioxy)benzylidene derivative related to **AnnH18**. 

**Scheme 2:**
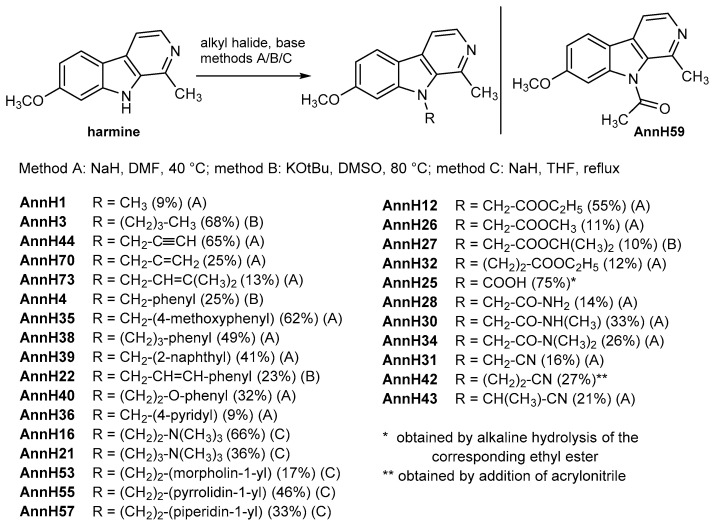
Harmine analogues prepared by *N*-acetylation (to give **AnnH59**) or *N*-alkylation. The yield as well as the respective *N*-alkylation method (A/B/C) are indicated in parentheses.

Further, we prepared a considerable number of *N*-alkylated products. This work was inspired by the above-mentioned work of Drung et al. [20] and reports describing 9-ethylharmine as promising DYRK1A inhibitor [24,32]; further, Cuny et al. [33] described a series on *N*-aminoalkyl derivatives, but tested them only on Haspin kinase and DYRK2. In order to obtain a clear insight into the structure–activity relationships in this subclass, we introduced small and large alkyl residues, arylalkyl residues of different length, as well as polar groups, either neutral, acidic or basic in nature. These *N*-alkylations were generally easy to perform, as shown by Cao et al. [34] and in a patent [35] for a couple of compounds before, but yields differed strongly depending on the alkylation agent. Finally, we established three different protocols for these conversions: (A) using sodium hydride as base in DMF at 40 °C, B) using potassium *tert*-butoxide as base in DMSO at 80 °C, and C) using sodium hydride as base in THF under reflux), which provided the target compounds (Scheme 2) in acceptable yields.

#### 2.1.2. De novo-Synthesis of Harmine Analogues

##### Harmine-Bauerine C hybrids—7,8-Dichloro Derivatives

As pointed out above, the unique binding mode (halogen bridge) of *ortho*-dichlorinated indoles and β-carbolines (Figure 1B) in the active site of kinases closely related to DYRK1A prompted us to explore this motif here as well. We hypothesized that, with this modification, DYRK1A affinity might be conserved, while MAO-A inhibition would hopefully be eliminated. Synthesis of the target compounds started with 1-oxo-β-carboline **AnnH65**, a compound obtained by dehydration of **1**, a precursor of our first total synthesis of bauerine C [36]. Compound **AnnH65** itself was of interest, since some 3-aza analogues thereof (pyridazino[4,5-b]indol-4-ones) were recently identified as potent DYRK1A inhibitors by Bruel et al. [37]. The ring A pyridone moiety in **AnnH65** was conveniently transformed into the 1-bromo derivative **AnnH52** by treatment with POBr_3_, using anisole as solvent and bromine scavenger [38], by which undesired ring bromination at ring C was successfully suppressed. The 1-methyl group was introduced by a chemoselective Pd-catalyzed cross-coupling with trimethylaluminum [39]. The 1-bromo substituent reacted exclusively in presence of both chlorine residues, and the target compound **AnnH63** was obtained in 92% yield.

**Scheme 3:**
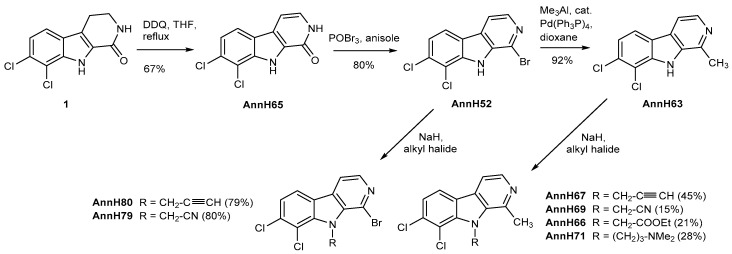
Synthesis of harmine-bauerine C hybrids and their *N*-alkylated analogues.

Since our initial work on *N^9^*-alkylated derivatives of harmine (see Section 2.1.1 and Section 2.2) clearly demonstrated a beneficial effect of small electron-withdrawing residues like cyanomethyl and related esters, we further performed *N*-alkylations with the respective alkyl halides on both the 1-bromo- and 1-methyl-7,8-dichloro-β-carboline (**AnnH52** and **AnnH63**) yielding products **AnnH66**, **AnnH67**, **AnnH69** as well as **AnnH79** and **AnnH80**; further aminopropyl derivative **AnnH71** was prepared for getting deeper insight into structure–activity relationships in this subtype of harmine analogues (Scheme 3).

##### Further Variations in Ring A

**Scheme 4:**
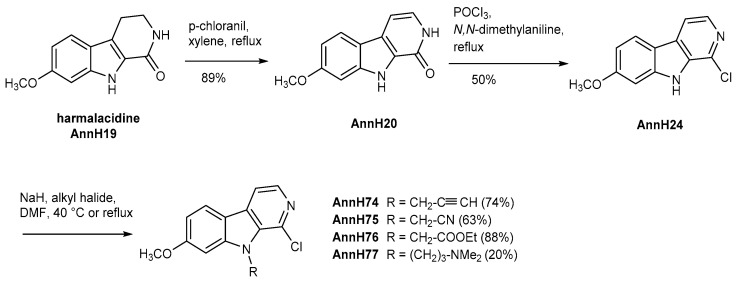
Synthesis of harmine analogues modified at position C-1 and subsequent *N*-alkylations.

Elimination/Reduction of the Basicity of N-2: In order to investigate the role of the nitrogen atom at position 2, we prepared two analogues of harmine lacking a 2-N with significant basicity. In a first modification, lactam-type alkaloid harmalacidine (**AnnH19**), readily available from 6-methoxytryptamine [40], was dehydrogenated with *p*-chloranil to give the ring A pyridone **AnnH20**. Our attempts to convert this 1-oxo-β-carboline into the 1-bromo compound using the POBr_3_/anisole couple (see Section 2.1.2) failed, since bromination at electron-rich ring C could not be suppressed here. Therefore, we performed a chlorination with POCl_3_, following published work [21,41], to receive the 1-chloro analogue **AnnH24** of harmine (Scheme 4). The electronegativity of the lactam oxygen in the one, and of the electronegative chlorine substituent in the other compound were expected to change the electronic properties of N-2 significantly.

In the same manner as done before with harmine (Section 2.1.1.) and the 1-bromo-7,8-dichloro analogue **AnnH52**, we introduced polar residues at N-9 using the established *N*-alkylation protocols, ending up with compounds **AnnH74**, **AnnH75**, **AnnH76**, and **AnnH77**.

**Scheme 5:**
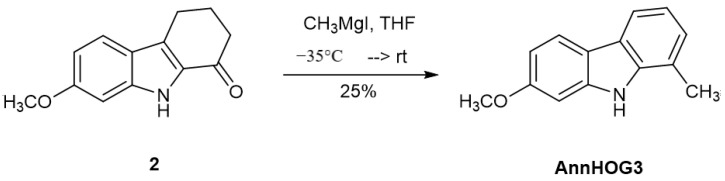
Synthesis of the 2-desaza analogue **AnnHOG3** of harmine.

Further, we prepared a 2-desaza analogue of harmine, represented by the disubstituted carbazole **AnnHOG3**. This compound was obtained from known tetrahydrocarbazol-1-one **2 [42]** through addition of methyllithium to the keto group. During work-up, dehydratisation and dehydrogenation took place to give the fully aromatic target compound in 25% yield (Scheme 5).

**Scheme 6:**
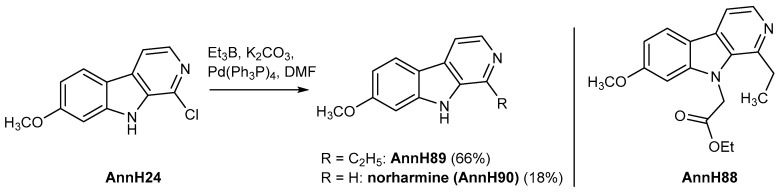
Synthesis of 1-ethyl analogues of harmine (and by-product norharmine).

Synthesis of 1-Ethyl Analogues of Harmine: Since even small changes in the size of a ligand of an enzyme may have significant influence on its affinity—for example, due to steric effects—we further prepared an analogue of harmine where the 1-methyl group was replaced by an ethyl group. Target compound **AnnH89** was obtained with a 66% yield from 1-chloro derivative **AnnH24** by Pd-catalyzed cross-coupling with triethylborane following the protocol we established for the synthesis of alkaloid 1-ethyl-β-carboline previously [39]. As a by-product, we obtained dechlorination product norharmine (**AnnH90**) at low yield. In an analogous manner, *N*-substituted 1-chloro compound **AnnH76** was coupled with triethylborane to give the ester **AnnH88** at 69% yield (Scheme 6).

##### Further Variations in Ring B: Replacement of NH by Ketone and Carbinol

As pointed out in the introduction of this manuscript, the NH functionality is important for the interaction of harmine with MAO-A, acting as a hydrogen bond donor for a water molecule, whereas a comparable interaction has not been reported for DYRK1A binding. Consequently, it was of interest to study the effect of replacing this NH group by a secondary alcohol (which is able to act as both a hydrogen bond donor and acceptor) and a keto group (which is exclusively a hydrogen bond acceptor) on the inhibitory activity on DYRK1A and MAO-A. This required a novel approach allowing the synthesis of appropriately substituted 2-azafluorenes.

**Scheme 7:**
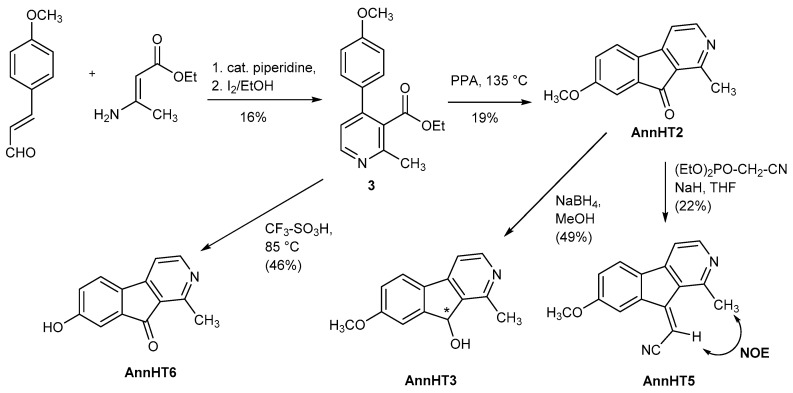
Synthesis of 9-desaza analogues of harmine.

The target 2-azafluorenone **AnnHT2** was obtained starting from 4-arylnicotinate **3**, which in turn was obtained by Hantzsch-type condensation [43] of 4-methoxycinnamaldehyde and ethyl 3-aminocrotonate, followed by dehydrogenation of the crude dihydropyridine intermediate with iodine. Cyclization to give the 2-azafluorenone **AnnHT2** was accomplished as demonstrated before for the 4-azafluorenone alkaloid onychine [44], by intramolecular acylation upon heating with polyphosphoric acid. Since the yield of this reaction was only 19%, we also examined a significantly stronger acid, trifluoromethanesulfonic acid. Here, a 2-azafluorenone was obtained in 46% yield, but the product **AnnHT6** was identified as the free phenol at C-7 (Scheme 7). Ketone **AnnHT2** was readily reduced to the racemic secondary alcohol **AnnHT3** with sodium borohydride. Unfortunately, diverse attempts to reduce the keto or carbinol function to a methylene group failed.

Since screening data of harmine derivatives bearing a cyanomethyl residue at N-9 were promising, we introduced a related cyanomethylene moiety into the obtained 9-desazaharmine scaffold. Horner–Wadsworth–Emmons olefination of ketone **AnnHT2** with diethyl cyanomethylphosphonate/NaH gave *E*-configured cyanomethylene product **AnnHT5** in 22% yield. The *E* configuration was confirmed by NOE experiments clearly indicating steric proximity of the olefinic methine hydrogen and the 1-methyl group (Scheme 7).

### 2.2. Pharmacological Testing

#### 2.2.1. In Vitro Enzyme Assays

All analogues of harmine described above were subjected to in vitro tests for inhibition of MAO-A and DYRK1A. Further, most compounds were tested for inhibition of the protein kinase CLK1, another member of the CMGC group of protein kinases, since DYRK1A inhibitors frequently also show strong inhibition of this kinase [26,27]. Optimization of the compounds, however, aimed predominantly at elimination of MAO-A-inhibitory activity while keeping DYRK1A inhibitory potency, and not at achieving high selectivity for DYRK1A over CLK1. The tests were performed as described by us before in detail [22]. Due to the very large number of test compounds (more than 60), a first screening was performed at a single inhibitor concentration of 1 µM. The results are presented in Table 1. Only outstanding compounds from this screening were subjected to further pharmacological characterization, and these results have been published by us before [22].

**Figure 3:**
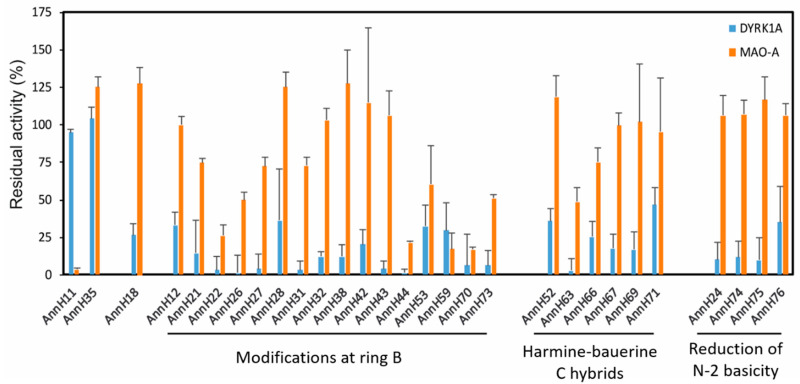
Inhibition of DYRK1A and MAO-A by selected harmine analogues. The diagram summarizes the screening results of the most relevant inhibitors from Table 1 to illustrate the different degrees of selectivity for DYRK1A. Shown are inhibitors that reduce DYRK1A activity by at least 50%. For comparison, **AnnH11** is included as an example for MAO-A selective inhibitor and **AnnH35** as a non-selective inhibitor. Columns reflect the residual enzyme activities of DYRK1A (blue) and MAO-A (orange) after incubation with the indicated compounds at a concentration of 1 µM (means and SD).

The values of inhibition of MAO-A and DYRK1A for the most relevant inhibitors (including standard deviations, SD) are presented in Figure 3.

As described in our recent publication [22], the most attractive compounds from this program were subjected to a comprehensive pharmacological characterization, and the main results are summarized in Table 2.

#### 2.2.2. Discussion of the Screening Results 

Results obtained for the alkaloids harmane and norharmine (**AnnH90**) clearly indicated the importance of the 7-methoxy group for DYRK1A inhibition, but also 7-hydroxy compound harmol was a potent DYRK1A inhibitor (and even more potent on CLK1), notably devoid of MAO-A inhibition, as shown by Balint et al. previously [21]. 1-Styryl analogue **AnnH18** was devoid of MAO-A inhibition, but at the cost of significantly reduced potency at DYRK1A.

Modifications at ring C: Bromination or chlorination at C-6 and/or C-8 eliminated DYRK1A inhibition completely. The monohalogenated products (including their *N*-butylated derivatives (**AnnH9**, **AnnH11**) retained significant MAO-A inhibition. This change in selectivity was exactly the opposite of what we intended to achieve. For the 7-aminoethyl ether **AnnH62**, we confirmed the published moderate MAO-A inhibitory potency [18] and found, not surprisingly, loss of kinase inhibition due to the known steric constraints at the small binding pocket of DYRK1A [19]. For this reason, we did not perform further modifications at C-7 with large substituents.

Modifications at N-9: Introduction of small (C_1_ to C_5_) alkyl/alkenyl/alkynyl residues preserved or even slightly improved DYRK1A inhibition, but led only to moderate reduction of MAO-A inhibition. Larger lipophilic residues (benzyl, arylalkyl) at N-9 typically reduced or eliminated both MAO-A and kinase inhibition, and the same holds for aminoalkyl residues. In contrast, small ester-containing residues derived from acetic or propionic acid and short cyanoalkyl groups led to significant reduction of MAO-A (and mostly also CLK1) inhibition, whereby DYRK1A inhibition was not affected. The free carboxylic acid **AnnH25**, however, showed an opposite shift in selectivity. This made aliphatic esters and even more nitriles at N-9 the most attractive modifications for the elimination of MAO-A inhibitory properties of harmine derivatives. The evidence gained from this first screening was fully confirmed for the cyanoalkyl derivatives **AnnH31** and **AnnH43**, as demonstrated by the IC_50_ values determined for MAO-A and DYRK1A inhibition [22] (Table 2).

Harmine-bauerine C hybrids: The ring A pyridone analogue (**AnnH65**) of harmine could not be analyzed properly due to its very poor solubility. The 1-bromo analogue **AnnH52** as well as its N-substituted derivatives **AnnH79/AnnH80** no longer inhibited MAO-A, but also lost DYRK1A inhibitory potency significantly (**AnnH52**) or almost completely. In contrast, the analogous 1-methyl-7,8-dichloro-β-carboline **AnnH63** was identified as a very potent DYRK1A inhibitor, but still showed significant MAO-A inhibition. Introduction of an aminoalkyl residue at N-9 in **AnnH71** expectedly reduces DYRK1A inhibition, whereas a cyanomethyl residue in **AnnH69** (and surprisingly also a propargyl group; **AnnH67**) resulted in very interesting compounds with significant DYRK1A inhibition and lack of activity on MAO-A. Docking studies aimed at the identification of the binding mode of **AnnH69** are presented in Section 2.3.2.

Investigations on the effect of a significant basicity of N-2 in harmine showed that replacing the pyridine ring A with a (dihydro)pyridone (**AnnH19**, **AnnH20**) leads to complete loss of inhibitory activity on all enzymes of interest. The same holds for the 2-desaza analogue **AnnHOG3**, clearly demonstrating the relevance of N-2 with sufficient basicity for the interaction with DYRK1A. In contrast, the 1-chloro analogue **AnnH24** of harmine was found to be a potent DYRK1A inhibitor, free of MAO-A-inhibitory activity. Similar effects were found for its *N*-cyanomethyl analogue **AnnH75** and the *N*-propargyl compound **AnnH74**; ester analogue **AnnH77** lost potency on DYRK1A, and 9-aminoalkyl derivative **AnnH77** expectedly was virtually inactive, once again demonstrating the detrimental effect of this group. Detailed pharmacological investigation (Table 2 and [22]) showed that **AnnH75** is the most attractive harmine modification from this project.

Exchanging the 1-methyl group in harmine by an ethyl group (also in combination with an ethoxycarbonylmethyl group at N-9 in **AnnH88**) did not lead to any beneficial changes in selectivity, but was accompanied by some loss of potency on DYRK1A. 

The relevance of the NH group in the central pyrrole ring of harmine was investigated by introduction of other polar groups at this position. Introduction of carbonyl, carbinol and cyanomethylene residues led to products with inferior selectivity and poor DYRK1A inhibition. Only ketone **AnnHT2** retained significant MAO-A inhibitory potency.

In conclusion, the following structure–activity relationships can be deduced from the results presented in Table 1 and Table 2:

1. The relevance of the main interactions of harmine with DYRK1A seen in the crystal structure [16] involving direct interactions with N-2 and the 7-methoxy group was confirmed here. Strong inhibition of DYRK1A requires the N-2 with significant basicity. The methoxy group at C-7 is a very well fitting substituent, larger ether groups at this position are not well tolerated. The methoxy group can be replaced by a chloro substituent, which most likely leads to a very similar binding geometry, involving a halogen bridge with the backbone in the hinge region (see Section 2.3.2), similar to previous reports on chlorinated β-carbolines bound to related kinases [25,26,27].
2. For DYRK1A inhibition, the methyl group at C-1 can be replaced by chlorine, but not by bromine.
3. Small alkyl groups as well as small polar groups (nitriles, esters, but not carboxylate, carboxamides and aminoalkyl groups) are tolerated at N-9. Replacement of N-9 by carbonyl or carbinol groups leads to a significant reduction of DYRK1A inhibition.
4. MAO-A-inhibitory activity is most effectively reduced by introduction of small, polar residues (which do not significantly reduce DYRK1A inhibition) at N-9. Further, the 1-chloro motif at C-1 appears beneficial for achieving selectivity for DYRK1A over MAO-A.
5. As expected, a clear dissection of DYRK1A inhibition from CLK1 inhibition could not be achieved despite the comprehensive structure variations presented here. A few β-carbolines (**AnnH21, AnnH26, AnnH27**) show some selectivity for CLK1, but on the other hand show insufficient selectivity over MAO-A.

### 2.3. X.ray Crystallography and Docking Studies

#### 2.3.1. Co-Crystal Structure of **AnnH75** with DYRK1A

**Figure 4:**
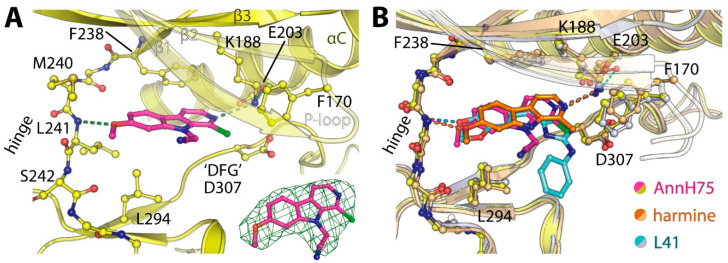
Crystal structure of DYRK1A in complex with **AnnH75**. (**A**) Detailed view of interaction between the inhibitor and the kinase. The inset shows an electron density map for the bound inhibitor. (**B**) Structural comparison between the binding modes of **AnnH75**, harmine (PDB 3ANR) and leucettine L41 (PDB 4AZE) revealed highly conserved binding modes of all inhibitors, albeit slight differences in orientation of the tricyclic core observed for **AnnH75**.

We next determined the complexed crystal structure of DYRK1A with **AnnH75** (PDB 4YU2) and analyzed the binding mode [45,46,47,48,49,50,51,52]. By comparison, **AnnH75** binds to the active site of DYRK1A in a very similar manner as described for harmine (PDB 3ANR) and L41 (PDB 4AZE) (Figure 4) with the pyridine N-2 moiety of **AnnH75** engaging in a direct H-bond to the salt bridge (Lys188-Glu203). Nevertheless, the tricyclic core of **AnnH75** was slightly shifted away from the catalytic salt bridge compared to harmine, hence resulting in a slightly longer distance between **AnnH75** N-2 to Lys188 nitrogen (3.3 Å) than the equivalent moiety in harmine (~3 Å) (Figure 4B). This may be due to the weaker basicity of the N-2 in **AnnH75** (due to the electronegative chlorine substituent at C-1) or to steric constraints of the cyanomethyl residue at position N-9. These effects might contribute to the slight loss in inhibitory activity of **AnnH75** compared to harmine (see Table 2). Notably, the cyanomethyl residue at N-9 did not make any direct or indirect interaction with DYRK1A in the structure, but it occupied a binding pocket located under the P-loop.

**Figure 5:**
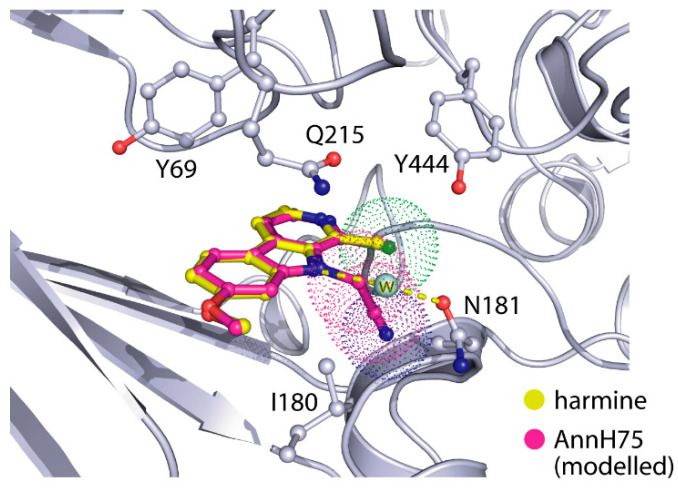
**AnnH75** (magenta) docked into the harmine (yellow)-MAO-A structure (PDB 2ZGX).

Docking **AnnH75** into the MAO-A structure (see Figure 1B) revealed that the cyanomethyl group is unfavorable, as this group would generate steric constraints within such a tight pocket where the harmine pyrrole N-9-H is located and forms a water-bridged hydrogen bond to Asn181 (Figure 5). Upon alkylation of N-9, the molecule is further missing the essential NH group for the formation of the hydrogen bond network. In addition, chloro decoration at C-1 may not be optimal for MAO-A binding either, since it might create some charge clashes with the hydroxyl group of Tyr444. Therefore, this model can explain the loss in affinity of **AnnH75** towards MAO-A.

#### 2.3.2. Additional Docking Studies

**Figure 6:**
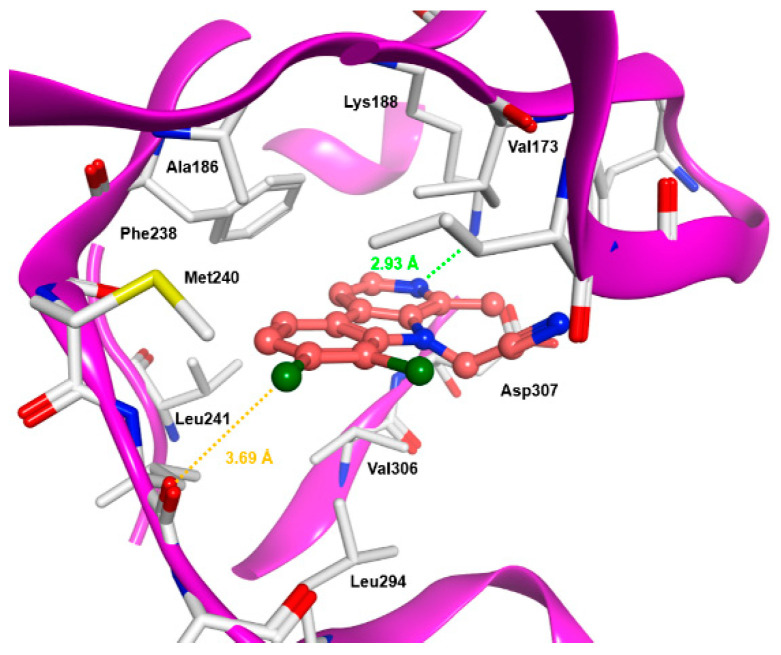
Harmine-bauerine C hybrid **AnnH69** docked into DYRK1A (PDB 4YU2). Carbon atoms in light brown, chlorine atoms in green, nitrogen atoms in blue, oxygen atoms in red.

We further performed docking studies on some other compounds that showed remarkable activity profiles. Harmine-bauerine C hybrid **AnnH69** was found to have a selectivity profile very similar to **AnnH75** (no inhibition of MAO-A, strong inhibition of DYRK1A and CLK1; see Table 1), but with slightly reduced inhibition of both kinases. Docking experiments suggested a binding pose in DYRK1A closely related to the one of **AnnH75**, with pyridine N-2 forming a hydrogen bridge with Lys144 and, as expected from the binding of our previously developed kinase inhibitors KH-CB19 and KH-CARB13 [25,26] (Figure 2B), an interaction of the chlorine substituent at C-7 with the backbone of Leu241 in the hinge region (Figure 6). Docking **AnnH69** with MAO-A did not give a favorable docking pose, most likely due to steric constraints of the *N*-cyanomethyl group.

**Figure 7:**
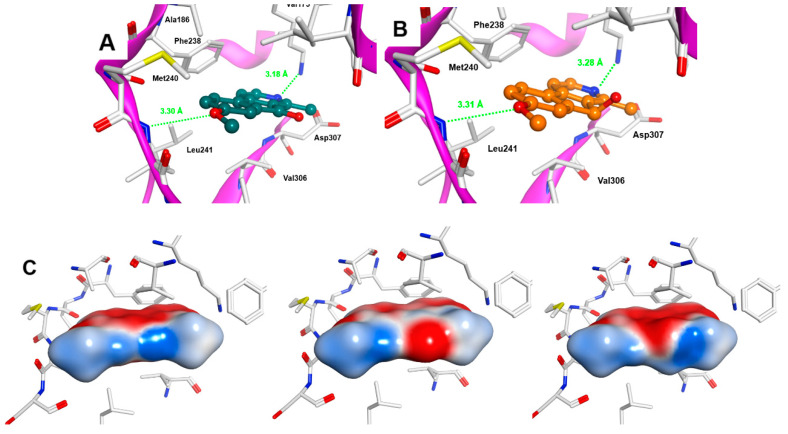
9-Desazaharmine analogues (**A**) **AnnHT2** and (**B**) **AnnHT3** (*S* enantiomer shown) docked into DYRK1A (PDB 4YU2; oxygen atoms in red, nitrogen atoms in blue). (**C**) Molecular electrostatic potentials of harmine (left), **AnnHT2** (center) and **AnnHT3** (right) (blue = electropositive, red = electronegative).

The 9-desazaharmine analogues **AnnHT2** (carbonyl group instead of NH) and **AnnHT3** (corresponding secondary alcohol) showed strongly reduced inhibition of DYRK1A (and CLK1), but still strong (**AnnHT2**) or medium (**AnnHT3**) inhibition of MAO-A (Table 1). Docking both compounds into DYRK1A (PDB 4YU2) suggested binding poses very similar to those of harmine (Figure 1A) and **AnnH75** (Figure 4) under involvement of N-2 and the 7-methoxy group (Figure 7A,B). Calculation of the molecular electrostatic potentials, however, showed unfavorable electronegative potentials for the desaza analogues, whereas harmine is electropositive around N-9 (Figure 7C). Most likely, this change in electrostatic potentials significantly reduces binding to the active site of DYRK1A.

**Figure 8:**
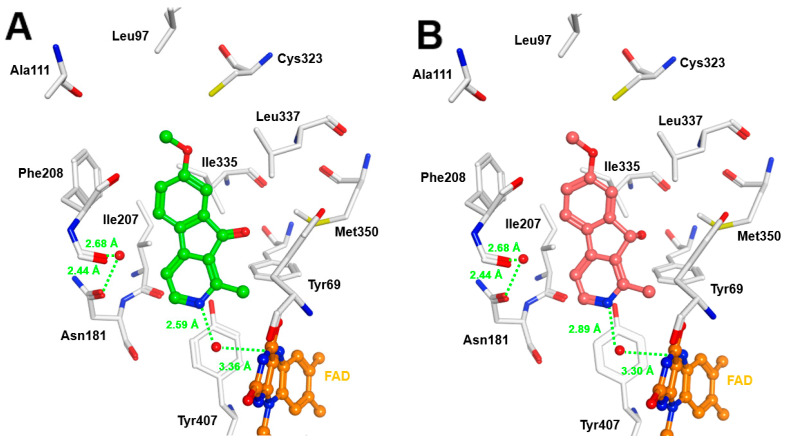
9-Desazaharmine analogues (**A**) **AnnHT2** (carbon atoms in green) and (**B**) **AnnHT3** (carbon atoms in brown; *S* enantiomer shown) docked into MAO-A (PDB 2Z5X). Oxygen atoms in red, nitrogen atoms in blue, water molecules shown as red balls.

Docking **AnnHT2** (Figure 8A) and **AnnHT3** (Figure 8B) into MAO-A (PDB 2Z5X) revealed binding poses similar to those of harmine (Figure 1B), with N-2 forming the same water-mediated hydrogen bond networks as harmine. The carbonyl (in **AnnHT2**) and carbinol (in **AnnHT3**) groups, however, are not oriented in the same direction as the 9-NH of harmine; rather, the molecules are oriented in an inverse manner, and do not form a second water-mediated hydrogen bridge network (with Ile207 and Asn181). This difference in orientation and different polar interactions might explain the moderate (**AnnHT2**) or stronger (**AnnHT3**) reduction of MAO-A inhibitory potency of these analogues.

## 3. Discussion

The protein kinase DYRK1A has important functions in neuronal development and cell cycle control, and has attracted large attention as a possible drug target for treatment of neurodegenerative processes, certain cancers, diabetes, and other diseases [2]. The β-carboline alkaloid harmine is a potent DYRK1A inhibitor, but it suffers from undesired strong inhibition of MAO-A, which would prevent application as a drug or as an investigative DYRK1A inhibitor in vivo. We synthesized more than 60 analogues of harmine, either by direct modification of the native alkaloid or by de novo synthesis of the β-carboline and related scaffolds in order to investigate structure–activity relationships for DYRK1A inhibition and to separate this desired activity from undesired MAO-A inhibition. Based on published crystal structures of harmine bound to DYRK1A and MAO-A, we modified all relevant functionalities in the molecule systematically, and identified a number of promising harmine analogues, which are characterized by optimized substituents at position N-9 (which preserve DYRK1A inhibition and eliminate MAO-A inhibition) and for DYRK1A binding beneficial moieties at C-1 (methyl or chlorine). From this series, **AnnH75** was identified as the most attractive DYRK1A inhibitor, which completely lacks MAO-A inhibition.

The binding mode of this inhibitor was elucidated by crystal structure analysis (PDB 4YU2), and docking experiments gave additional insight into binding of this and related compounds to DYRK1A and MAO-A. The chemical space in the field of β-carbolines was widely explored here, but most recent achievements from our research group on the introduction of additional substituents into ring A [53,54] might even open additional opportunities for optimization. 

**AnnH75** is a valuable chemical tool for further investigation of the physiological role of DYRK1A, and the comprehensive evidence on structure–activity relationships from this project should further inspire future attempts in the development of DYRK1A inhibitors.

## 4. Materials and Methods

### 4.1. Enzyme Assays

Inhibitors were dissolved in DMSO at a concentration of 10 mM and stored at 20 °C. Working solutions were prepared to achieve a final concentration of 1 µM in the enzyme assays.

Recombinant kinases were purified from *E. coli* as GST (glutathione S-transferase) fusion constructs by affinity adsorption to glutathione-Sepharose. The expression constructs for GST-DYRK1A-ΔC (containing amino acids 1–499 of rat DYRK1A) and GST-CLK1cat (containing amino acids 141–484 of human CLK1) have been described before [22]. Catalytic activity of the kinases was measured with the help of the Kinase-GLO^TM^ Luminescent Assay from Promega. Assays were performed in a total volume of 10 μL in kinase buffer (25 mM Hepes pH 7.4, 0.5 mM DTT, 5 mM MgCl_2_, 5 μM ATP) with appropriate peptide substrates (20 μM DYRKtide for the DYRKs and 100 μM DYRKtide for HIPK2, RRRFRPASPLRGPPK; or 100 μM RS peptide for CLK1, GRSRSRSRSR). Kinases amounts in the assays were adjusted so that approximately 90% of the ATP in the assay was consumed in control samples without inhibitor. Kinase reactions were incubated at room temperature for 30 min before 10 μL Kinase-GLO reagent was added for luminometric measurement of the ATP that was not consumed by the kinases. After incubation at room temperature for an additional 10 min, luminescence was recorded for 1 s (Berthold Orion Microplate Luminometer). ATP consumption in the control samples without inhibitor was set as 100% catalytic activity, and the inhibitory effects of the test compounds were calculated relative to this value.

Catalytic activity of monoamine oxidase A (MAO-A) was measured by using the luminescence-based MAO-GLO^TM^ Assay from Promega. Recombinant MAO-A converts a luminogenic MAO substrate into luciferin, which is, in turn, luminometrically detected in a luciferase reaction. The amount of light produced is directly proportional to MAO activity. Assays were run at room temperature for 1 h with 12 µU human recombinant MAO-A and 25 µM MAO-A substrate in a total volume of 20 µL. Inhibitory effects of the tested compounds were calculated relative to the activity of control reactions that were run in the absence of inhibitors.

Kinase and MAO-A assays were run with duplicate measurements, and the assay results in Table 1 and Figure 3 represent means of *n* = 3 experiments except for the following values: DYRK1A results represent *n* = 5 for **AnnH43**, **AnnH44**, *n* = 7 for **AnnH31**, MAO-A results represent *n* = 4 for **AnnH38**, **AnnH43** and CLK1 results represent *n* = 4 for **AnnH18**, **AnnH24**, **AnnH38**, **AnnH43**, *n* = 5 for **AnnH12** and *n* = 7 for **AnnH31** and *n* = 2 for a number of compounds as indicated in the footnotes to Table 1.

### 4.2. Crystallization and Structure Determination Protein Production, Crystallization, Data Collection and Structure Determination

The recombinant DYRK1A kinase domain protein was expressed and purified as previously described [45]. The N-termial His_6_ tag was removed, and the resulting cleaved protein was buffered in 50 mM HEPES pH 7.5, 500 mM NaCl and 5 mM DTT. The protein was concentrated to 15.6 mg/mL and was mixed with 1 mM **AnnH75**. Crystallization was performed using the sitting drop vapor diffusion method at 4 °C and the reservoir solution containing 31% PEG 400, 0.2 M Li_2_SO_4_ and 0.1 M Tris pH 9.0. Diffraction data were collected at Diamond Light Source, beamline I04-1, and were processed and scaled with XDS [46] and Scala from CCP4 suite [47], respectively. The structure determination was achieved by molecular replacement using Phaser [48] and the coordinates of DYRK1A structure [45] as a search model. Manual model building was performed in COOT [49], and the structure was refined using Refmac [50] with a TLS model calculated from TLSMD server [51]. The final model was verified for its geometric correctness with MOLPROBITY [52]. The data collection and refinement statistics are summarized in Table 3.

### 4.3. Computational Methods

The GLIDE software version 9.3 was used for docking, whereas the calculation of all molecular descriptors and the analysis of the docking results were carried out in MOE20012.10 (Chemical Computing Group). All ligands were constructed using MOE2012.10 and energy minimized using the MMFF94 force field with a convergence criteria of 0.1 kcal/mol. All compounds were first docked into the binding pocket of the X-ray structure of DYRK1A (PDB code 3ANR complexed with harmine and which represents the active form of DYRK1A), CLK1 (PDB code 2VAG complexed with KH-CB19, which represents the active form of CLK1), and MAO-A (PDB code 2Z5X complexed with harmine). The cocrystallized inhibitor was defined as centre of the enclosing box used for docking with a radius of 15 Å).

For docking into DYRK1A, two protein hydrogen bonds were defined, which are observed in the DYRK1A-harmine crystal structure: one to Leu241, which is part of the hinge region, and one to Lys188. No water molecules were considered for ligand docking. To test whether the used docking protocol is suitable for DYRK1A docking, the cocrystallized inhibitor was redocked into DYRK1A. Using GlideScore as scoring function, an RMSD value of 0.39 Å was derived for the top-ranked docking pose of harmin. For all docked inhibitors, the GlideScore was calculated and analyzed.

During the course of the project, we were able to cocrystallized **AnnH75** with DYRK1A (PDB code 4YU2) and confirmed the predicted docking pose of AnnH75 (RMSD 0.22–0.26 Å, in the four chains of 4YU2). All studied compounds were then also docked to 4YU2 and similar docking poses as for 3ANR were obtained.

Docking to CLK1 was carried out using the crystal structure in complex with the small molecule inhibitor KH-CB19 which has a similar size and shape as harmine. Two hydrogen bonds were defined similar as in case of DYRK1A: one to the hinge region residue Leu244 and one to Lys191. To test whether the used docking protocol is suitable for CLK1 docking, the cocrystallized inhibitor was redocked into the ATP binding pocket. Using GlideScore as scoring function, an RMSD value of 0.61 Å was derived for the top-ranked docking pose of KH-CB19. For all docked inhibitors, the GlideScore was calculated and analyzed.

For docking into MAO-A, the cofactor and most of the water molecules were removed. A total of seven water molecules, found inside the binding pocket of MAO-A, were considered for docking. To test the applicability of the docking tool, a control docking was carried out using the cocrystallized inhibitor harmin. Using GlideScore as scoring function, an RMSD value of 0.94 Å was derived for the top-ranked pose of harmin. For all compounds under study, the GlideScore was calculated and analyzed.

To calculate the molecular electrostatic potential (MEP) of the studied inhibitors, ESP-AM1 partial charges were calculated using program MOE2012.10. The electrostatic potential was calculated using the MM-GBSA option and displayed on the van-der-Waals surface of the inhibitors.

### 4.4. Chemistry

#### 4.4.1. General

All chemicals and solvents were used as purchased from commercial services (Sigma-Aldrich, Acros, Alfa Aesar) without further purification. The progress of all reactions was monitored by TLC on Machery nagel polygram sil plates G/UV_254_ (0.2 mm, 40 × 80 mm). Flash column chromatography was performed on Merck silica gel Si 60 (40–63 µm). NMR spectra were recorded on a Jeol GSX 400 or Jeol JNMR-GX 500 (Jeol, Peabody, MA, USA), using TMS as internal standard. Chemical shifts are reported in ppm and given in *δ* units. Coupling constants are given in Hertz. The spectra were recorded at room temperature. Mass spectra (electron ionization, EI, 70 eV) were recorded using a Hewlett Packard 5989A mass spectrometer with a 59,980 B particle beam LC/MS interface (Agilent Technologies, PAO Alto, CA, USA) or Thermo Finnigan MAT95 mass spectrometer (Thermo Fischer Scientific, Waltham, MA, USA). Mass spectra (APCI) were recorded using a ABSciex API 2000 LC/MS/MS (AB SCIEX, Foster City, CA, USA). Mass spectra (ESI) was recorded using a Thermo Finnigan LTQ FT Ultra (Thermo Fischer Scientific, Waltham, MA, USA). HRMS (EI) were performed using a Jeol JMS GCmate II (Jeol, Peabody, MA, USA) or HRMS (ESI) using a Thermo Finnigan LTQ FT (Thermo Electron Corporation, Waltham, MA, USA). IR spectra were recorded as KBr disks on a Perkin Elmer FT-IR Parago 1000 (Perkin Elmer, Waltham, MA, USA) or JASCO FT/IR-410 (Jasco, Easton, PA, USA). Melting points were determined with a Büchi B-540 apparatus (Büchi, Flawil, Switzerland) and are uncorrected. Purity of all compounds was determined with a HP Agilent 1100 HPLC (diode array detector, Agilent Technologies, Waldbronn, Germany) equipped with an Agilent Poroshell column (120 EC-C18; 3.0 × 100 mm, 2.7 microns) using following conditions: eluent acetonitrile/water/THF 8:2:0.1 or 6:4:0.1, flow rate: 0.9 mL/min, column temperature 50 °C, detection at 210 nm and 254 nm, injection of 5 µL of 100 µg/mL solutions of the compound. Microwave-assisted reactions were conducted using a single-mode microwave reactor Discover SP (300 W, CEM, Matthews, NC, USA).

#### 4.4.2. Synthesis of Compounds

General procedure A for the *N*-alkylation of varios β-carbolines with using of sodium hydride as base in DMF. To the appropiate β-carboline in dry DMF sodium hydride (60% in mineral oil) was added. The mixture was stirred at 40 °C for 20 min and the appropriate alkyl halide was added. The mixture was stirred at 40 °C for the specified time period and then over night at room temperature. The following detailed procedure for isolation of **AnnH44** is representative for the preparation of **AnnH44**, **AnnH31**, **AnnH70**, **AnnH73**, **AnnH35**, **AnnH36**, **AnnH39**, **AnnH40**, **AnnH38**, **AnnH12**, **AnnH26**, **AnnH32**, **AnnH30**, **AnnH28**, **AnnH34**, **AnnH43**, **AnnH74**, **AnnH75**, **AnnH76**, **AnnH80**, **AnnH79**, **AnnH67**, **AnnH69** and **AnnH66**.

General procedure B for the *N*-alkylation of β-carbolines using potassium *tert*-butoxide as base in DMSO. The appropriate β-carboline and potassium *tert*-butoxide were dissolved in anhydrous DMSO and stirred for 30 min at 80 °C. The reaction mixture was cooled to room temperature and then the appropriate alkyl halide was added and the mixture stirred at 80 °C for the specified time period and then cooled to room temperature. After quenching with cold aqueous ammonia solution (10%, 10 mL), the mixture was treated with water until a solid was precipitated. The aqueos layer was extracted with ethyl acetate (3 × 50 mL). The combined organics were dried over sodium sulfate and evaporated to dryness. The residue was purified by column chromatography (silica gel, iso-hexane/ethyl acetate/ethanol 2:2:1) to afford the corresponding alkylated β-carbolines **AnnH9**, **AnnH11**, **AnnH14**, **AnnH3**, **AnnH4**, **AnnH22** and **AnnH27**.

General procedure C for the *N*-alkylation of β-carbolines using sodium hydride as base in THF. A suspension of the appropriate β-carboline and sodium hydride (60% in mineral oil) in THF was stirred for 20 min, then the appropriate alkyl halide was added and the mixture was stirred under reflux for 65 h. The solution was allowed to cool to room temperature and water (10 mL) was added. The organic layer was separated and the aqueos layer was extracted with DCM (3 × 25 mL). The combined organic layers were dried over sodium sulfate and evaporated to dryness. The residue was purified by column chromatography (silica gel, iso-hexane/ethyl acetate/ethanol/triethylamine 2:2:1 +1%) to afford the corresponding alkylated β-carbolines **AnnH16**, **AnnH21**, **AnnH53**, **AnnH55**, **AnnH57**, **AnnH71** and **AnnH77**.

*7-Methoxy-1-methyl-9-(prop-2-yn-1-yl)-9H-pyrido[3,4-b]indole* (**AnnH44**). Prepared from harmine (153 mg, 0.720 mmol), sodium hydride (41 mg, 1.0 mmol), dry DMF (5.0 mL) and propargyl bromide (80% wt in toluene, 0.080 mL, 0.72 mmol). The mixture was stirred at 40 °C for 2 h. After treatment with a saturated aqueous solution of sodium carbonate (10 mL) and water (5 mL) the mixture was extracted with ethyl acetate (5 × 20 mL). The combined organic layers were dried over magnesium sulfate and evaporated to dryness. The residue was purified by column chromatography (silica gel, iso-hexane/ethyl acetate/ethanol 2:2:1) to afford **AnnH44** as a light brown solid (118 mg, 65%). m.p. 146–147 °C; ^1^H-NMR (400 MHz, CDCl_3_) *δ* = 8.31 (d, *J* = 5.2 Hz, 1 H, 3-H), 7.96 (d, *J* = 9.0 Hz, 1 H, 5-H), 7.71 (d, *J* = 5.2 Hz, 1 H, 4-H), 6.94–6.88 (m, 2 H, 6-H, 8-H), 5.18 (d, *J* = 2.5 Hz, 2 H, 1’-H), 3.94 (s, 3 H, OCH_3_), 3.10 (s, 3 H, 1-CH_3_), 2.35 (t, *J* = 2.5 Hz, 1 H, 3′-H); ^13^C-NMR (100 MHz, CDCl_3_) *δ* = 161.1, 142.7, 141.0, 139.1, 135.0, 129.7, 122.5, 115.4, 112.3, 109.5, 93.2, 78.5, 73.5, 55.7, 34.7, 23.0; IR (KBr) $\tilde{\nu }$ (cm^−1^) = 3431, 3064, 2119, 1615, 1171; MS (EI) *m/z* (relative intensity, %) = 250 [M^+**•**^] (100), 211 (60), 196 (8), 168 (21); HRMS (EI) calcd. for C_16_H_14_N_2_O: 250.1106; found 250.1107; purity: 100% (λ = 210 nm), 100% (λ = 254 nm) (by HPLC).

*8-Bromo-9-butyl-7-methoxy-1-methyl-9H-pyrido[3,4-b]indole* (**AnnH9**). Prepared from 8-bromo-7-methoxy-1-methyl-9*H*-pyrido[3,4-b]indole (**AnnH5_3**) (48 mg, 0.17 mmol), potassium *tert*-butoxide (22 mg, 0.20 mmol), dry DMSO (5 mL) and *n*-butyl iodide (0.10 mL, 0.88 mmol). The mixture was stirred at 80 °C for 3 h. Beige solid (34 mg, 60%). m.p. 90–96 °C; ^1^H-NMR (500 MHz, CDCl_3_) *δ* = 8.32 (d, *J* = 5.2 Hz, 1 H, 3-H), 7.98 (d, *J* = 8.5 Hz, 1 H, 5-H), 7.71 (d, *J* = 5.2 Hz, 1 H, 4-H), 6.93 (d, *J* = 8.5 Hz, 1 H, 6-H), 5.10–4.90 (m, 2 H, 1′-H), 4.02 (s, 3 H, OCH_3_), 3.03 (s, 3 H, 1-CH_3_), 1.72–1.63 (m, 2 H, 2′-H), 1.38–1.24 (m, 2 H, 3′-H), 0.93 (t, *J* = 7.4 Hz, 3 H, 4′-H); ^13^C-NMR (100 MHz, CDCl_3_) *δ* = 156.8, 141.6, 140.0, 138.9, 137.2, 129.5, 120.8, 119.2, 111.9, 105.7, 93.9, 57.2, 45.3, 33.7, 24.6, 19.5, 13.9; IR (KBr) $\tilde{\nu }$ (cm^−1^) = 3422, 2926, 1653, 1026; MS (EI) *m/z* (relative intensity, %) = 348 (54), 346 [M^+**•**^] (56), 307 (98), 305 (100), 225 (10), 57 (26); HRMS (EI) calcd. for C_17_H_19_N_2_OBr: 346.0681; found 346.0691; purity: 100% (λ = 210 nm), 100% (λ = 254 nm) (by HPLC).

*9-Butyl-8-chloro-7-methoxy-1-methyl-9H-pyrido[3,4-b]indole* (**AnnH11**). Prepared from 8-chloro-7-methoxy-1-methyl-9*H*-pyrido[3,4-b]indole (**AnnH6_1**) (54 mg, 0.22 mmol), potassium *tert*-butoxide (27 mg, 0.24 mmol), dry DMSO (5 mL) and *n*-butyl iodide (0.13 mL, 1.1 mmol). The mixture was stirred at 80 °C for 4 h. Beige solid (35 mg, 53%). m.p. 88–90 °C; ^1^H-NMR (500 MHz, DMSO-*d_6_*) *δ* = 8.24 (d, *J* = 5.1 Hz, 1 H, 3-H), 8.20 (d, *J* = 8.6 Hz, 1 H, 5-H), 7.95 (d, *J* = 5.1 Hz, 1 H, 4-H), 7.17 (d, *J* = 8.6 Hz, 1 H, 6-H), 4.88 (t, *J* = 7.8 Hz, 2 H, 1′-H), 3.98 (s, 3 H, OCH_3_), 2.95 (s, 3 H, 1-CH_3_), 1.65 (p, *J* = 7.7 Hz, 2 H, 2′-H), 1.31–1.24 (m, 2 H, 3′-H), 0.88 (t, *J* = 7.4 Hz, 3 H, 4′-H); ^13^C-NMR (100 MHz, DMSO-*d_6_*) *δ* = 150.2 135.8, 133.2, 132.2, 130.7, 123.2, 115.3, 112.6, 106.9, 100.8, 98.3, 51.4, 40.3, 33.5, 18.2, 13.5, 8.1; IR (KBr) $\tilde{\nu }$ (cm^−1^) = 3426, 2954, 1623, 1078; MS (EI) *m/z* (relative intensity, %) = 304 (17), 302 [M^+**•**^] (48), 261 (32), 259 (100), 246 (6), 244 (10), 218 (4), 216 (13); HRMS (EI) calcd. for C_17_H_19_N_2_OCl: 302.1186; found 302.1190; purity: 100% (λ = 210 nm), 100% (λ = 254 nm) (by HPLC).

*9-Butyl-6-chloro-7-methoxy-1-methyl-9H-pyrido[3,4-b]indole* (**AnnH14**). Prepared from 6-chloro-7-methoxy-1-methyl-9*H*-pyrido[3,4-b]indole (**AnnH6_3_2**) (53 mg, 0.21 mmol), potassium *tert*-butoxide (31 mg, 0.27 mmol), dry DMSO (5 mL) and *n*-butyl iodide (0.13 mL, 1.1 mmol). The mixture was stirred at 80 °C for 2 h. Beige solid (35 mg, 54%). m.p. 133–137 °C; ^1^H-NMR (400 MHz, DMSO-*d_6_) δ* = 8.34 (s, 1 H, 5-H), 8.19 (d, *J* = 5.2 Hz, 1 H, 3-H), 7.94 (d, *J* = 5.2 Hz, 1 H, 4-H), 7.37 (s, 1 H, 8-H), 4.58 (t, *J* = 7.5 Hz, 2 H, 1′-H), 4.02 (s, 3 H, OCH_3_), 2.95 (s, 3 H, 1-CH_3_), 1.72–1.68 (m, 2 H, 2′-H), 1.37–1.31 (m, 2 H, 3′-H), 0.92 (t, *J* = 7.4 Hz, 3 H, 4′-H); ^13^C-NMR (100 MHz, DMSO-*d_6_*) *δ* = 150.7, 136.7, 136.5, 133.5, 130.1, 123.2, 117.9, 109.9, 109.6, 108.1, 89.6, 52.1, 35.5, 28.0, 18.6, 15.0, 9.3; IR (KBr) $\tilde{\nu }$ (cm^−1^) = 3443, 3048, 1622, 1040; MS (EI) *m/z* (relative intensity, %) = 304 (14), 302 [M^+**•**^] (30), 261 (34), 259 (100), 246 (4), 244 (8), 218 (2), 216 (14); HRMS (EI) calcd. for C_17_H_19_N_2_OCl: 302.1186; found 302.1193; purity: 100% (λ = 210 nm), 93% (λ = 254 nm) (by HPLC).

*1-(7-Methoxy-1-methyl-9H-pyrido[3,4-b]indol-9-yl)ethan-1-one* (*N*^9^-acetylharmine; **AnnH59**). Harmine (103 mg, 0.484 mmol) and piperonal (160 mg, 1.07 mmol) in 3.0 mL of acetic anhydride were stirred at reflux for 3 h and at room temperature for 2 h. The reaction was cooled to room temperature and then treated with 60 mL of ice water. The pH was adjusted to 7–8 with a saturated aqueous solution of sodium hydrogen carbonate. The mixture was extracted with ethyl acetate (3 × 150 mL). The combined organic layers were dried over sodium sulfate and evaporated to dryness. The residue was purified by column chromatography (silica gel, iso-hexane/ethyl acetate/ethanol 2:2:1) to afford **AnnH59** as a beige solid (38 mg, 31%). m.p. 107–108 °C; ^1^H-NMR (400 MHz, CDCl_3_) *δ* = 8.50 (d, *J* = 5.1 Hz, 1 H, 3-H), 7.89 (d, *J* = 8.6 Hz, 1 H, 5-H), 7.63 (d, *J* = 5.1 Hz, 1 H, 4-H), 7.59 (d, *J* = 2.2 Hz, 1 H, 8-H), 7.00 (dd, *J* = 8.6, 2.2 Hz, 1 H, 6-H), 3.94 (s, 3 H, OCH_3_), 2.73 (s, 3 H, CO-CH_3_), 2.69 (s, 3 H, 1-CH_3_); ^13^C-NMR (100 MHz, CDCl_3_) *δ* = 170.6, 161.6, 145.8, 143.6, 141.8, 134.8, 134.2, 122.1, 117.7, 111.7, 111.1, 99.8, 55.8, 27.1, 24.7; IR (KBr) $\tilde{\nu }$ (cm^−1^) = 3443, 2979, 1702, 1626, 1170; MS (ESI) *m/z =* 255 [M^+^+ H], 213; HRMS (ESI) calcd. for C_15_H_14_N_2_O_2_ +H^+^: 255.1133; found 255.1133; purity: 99% (λ = 210 nm), 99% (λ = 254 nm) (by HPLC).

*9-Butyl-7-methoxy-1-methyl-9H-pyrido[3,4-b]indole* (**AnnH3**). Prepared from harmine (102 mg, 0.481 mmol), potassium *tert*-butoxide (58 mg, 0.52 mmol), dry DMSO (9 mL) and *n*-butyl iodide (0.27 mL, 2.4 mmol). The mixture was stirred at 80 °C for 2 h. Beige solid (85 mg, 68%). The analytical data were in accordance with those published in ref. [34].

*7-Methoxy-1-methyl-9-(prop-2-yn-1-yl)-9H-pyrido[3,4-b]indole* (**AnnH70**). Prepared from harmine (104 mg, 0.491 mmol), sodium hydride (24 mg, 0.60 mmol), dry DMF (2.5 mL) and allyl bromide (0.040 mL, 0.50 mmol). The mixture was stirred at 40 °C for 7 h. Beige solid (30 mg, 25%). m.p. 95–96 °C; ^1^H-NMR (500 MHz, CDCl_3_) *δ* = 8.29 (d, *J* = 5.2 Hz, 1H, 3-H), 7.99 (d, *J* = 8.7 Hz, 1 H, 5-H), 7.74 (d, *J* = 5.2 Hz, 1 H, 4-H), 6.90 (dd, *J* = 8.6, 2.2 Hz, 1 H, 6-H), 6.79 (d, *J* = 2.1 Hz, 1 H, 8-H), 6.09 (ddt, *J* = 17.2, 10.4, 4.0 Hz, 1 H, 2′-H), 5.20–5.16 (m, 1 H, 3′-H), 5.10–5.08 (m, 2 H, 1′-H), 4.83–4.79 (m, 1 H, 3′-H), 3.93 (s, 3 H, OCH_3_), 2.98 (s, 3 H, 1-CH_3_); ^13^C-NMR (100 MHz, CDCl_3_) *δ* = 170.0, 143.2, 140.9, 138.5, 135.6, 133.2, 129.3, 122.3, 116.5, 115.1, 112.2, 109.0, 93.3, 55.6, 46.9, 22.9; IR (KBr) $\tilde{\nu }$ (cm^−1^) = 3443, 2957, 1618, 1173; MS (APCI) *m/z =* 253 [M^+^+ H], 212; HRMS (ESI) calcd. for C_16_H_16_N_2_O + H^+^: 253.1338; found 253.1341; purity: 97% (λ = 210 nm), 100% (λ = 254 nm) (by HPLC).

*7-Methoxy-1-methyl-9-(3-methylbut-2-en-1-yl)-9H-pyrido[3,4-b]indole* (**AnnH73**). Prepared from harmine (107 mg, 0.505 mmol), sodium hydride (23 mg, 0.56 mmol), dry DMF (3 mL) and 3,3-dimethylallyl bromide (0.055 mL, 0.47 mmol). The mixture was stirred at 40 °C for 4 h. Beige solid (18 mg, 14%). m.p. 102–104 °C; ^1^H-NMR (500 MHz, CDCl_3_) *δ* = 8.24 (d, *J* = 5.2 Hz, 1 H, 3-H), 7.94 (d, *J* = 8.6, 1 H, 5-H), 7.69 (d, *J* = 5.2 Hz, 1 H, 4-H), 6.85 (dd, *J* = 8.6, 2.2 Hz, 1 H, 6-H), 6.78 (d, *J* = 2.1 Hz, 1 H, 8-H), 5.22–5.18 (m, 1 H, 2′-H), 5.08–5.06 (m, 2 H, 1′-H), 3.90 (s, 3 H, OCH_3_), 2.96 (s, 3 H, 1-CH_3_), 1.86 (d, *J* = 1.3 Hz, 3 H, C(CH_3_)_2_), 1.69 (d, *J* = 1.3 Hz, 3 H, C(CH_3_)_2_); ^13^C-NMR (125 MHz, CDCl_3_) *δ* = 160.8, 143.1, 140.9, 138.3, 135.5, 134.5, 129.3, 122.3, 121.8, 115.3, 112.2, 108.9, 93.4, 55.6, 43.4, 25.5, 23.3, 18.4; IR (KBr) $\tilde{\nu }$ (cm^−1^) = 3433, 2928, 1620, 1255; MS (APCI) *m/z =* 281 [M^+^+ H], 213; HRMS (ESI) calcd. for C_18_H_20_N_2_O + H^+^: 281.1653; found 281.1650; purity: 100% (λ = 210 nm), 100% (λ = 254 nm) (by HPLC).

*9-Benzyl-7-methoxy-1-methyl-9H-pyrido[3,4-b]indole* (**AnnH4**). Prepared from harmine (101 mg, 0.476 mmol), potassium *tert*-butoxide (58 mg, 0.52 mmol), dry DMSO (10 mL) and benzyl bromide (0.30 mL, 2.4 mmol). The mixture was stirred at 80 °C for 4 h. Beige solid (36 mg, 25%). The analytical data were in accordance with those published in ref. [34].

*7-Methoxy-9-(4-methoxybenzyl)-1-methyl-9H-pyrido[3,4-b]indole* (**AnnH35**). Prepared from harmine (150 mg, 0.706 mmol), sodium hydride (32 mg, 0.82 mmol), dry DMF (5.0 mL) and 4-methoxybenzyl chloride (0.10 mL, 0.70 mmol). The mixture was stirred at 40 °C for 3 h. Beige solid (145 mg, 62%). m.p. 164–166 °C; ^1^H-NMR (500 MHz, CDCl_3_) *δ* = 8.30 (d, *J* = 5.2 Hz, 1 H, 3-H), 8.01 (d, *J* = 8.6 Hz, 1 H, 5-H), 7.77 (d, *J* = 5.2 Hz, 1 H, 4-H), 6.95–6.87 (m, 3 H, 6-H, 3″/5″-H), 6.82–6.78 (m, 2 H, 2″/6″-H), 6.77 (d, *J* = 2.1 Hz, 1 H, 8-H), 5.66 (s, 2 H, 1′-H), 3.85 (s, 3 H, OCH_3_), 3.75 (s, 3 H, 4″-OCH_3_), 2.86 (s, 3 H, 1-CH_3_); ^13^C-NMR (125 MHz, CDCl_3_) *δ* = 161.0, 158.9, 143.5, 141.0, 138.6, 135.7, 129.8, 129.3, 126.6 (2 C), 122.4, 115.1, 114.4 (2 C), 112.3, 109.2, 93.3, 55.6, 55.3, 47.7, 23.1; IR (KBr) $\tilde{\nu }$ (cm^−1^) = 3045, 1619, 1027; MS (EI) *m/z* (relative intensity, %) = 332 [M^+**•**^] (8), 121 (100); HRMS (EI) calcd. for C_21_H_20_N_2_O_2_: 332.1525; found 332.1524; purity: 100% (λ = 210 nm), 100% (λ = 254 nm) (by HPLC).

*7-Methoxy-1-methyl-9-(3-phenylpropyl)-9H-pyrido[3,4-b]indole* (**AnnH38**). Prepared from harmine (151 mg, 0.712 mmol), sodium hydride (29 mg, 0.72 mmol), dry DMF (5.0 mL) and 1-bromo-3-phenylpropane (0.11 mL, 0.73 mmol). The mixture was stirred at 40 °C for 4 h. Beige solid (114 mg, 49%). The analytical data were in accordance with those published in ref. [34].

*7-Methoxy-1-methyl-9-(2-naphthylmethyl)-9H-pyrido[3,4-b]indole* (**AnnH39**). Prepared from harmine (150 mg, 0.709 mmol), sodium hydride (28 mg, 0.71 mmol), dry DMF (5.0 mL) and 2-(bromomethyl)naphthalene (0.159 mg, 0.716 mmol). The mixture was stirred at 40 °C for 4 h. Beige solid (102 mg, 41%). m.p. 143 °C; ^1^H-NMR (500 MHz, CD_2_Cl_2_) *δ* = 8.27 (d, *J* = 5.2 Hz, 1 H, 3-H), 8.05 (d, *J* = 8.6 Hz, 1 H, 5-H), 7.82–7.80 (m, 3 H, 4-H, 4′′-H, 5′′-H), 7.60 (d, *J* = 7.8 Hz, 1 H, 8′′-H), 7.48–7.37 (m, 2 H, 6′′-H, 7′′-H), 7.33 (s, 1 H, 1′′-H), 7.26 (dd, *J* = 8.5, 1.8 Hz, 1 H, 3′′-H), 6.91 (dd, *J* = 8.6, 2.2 Hz, 1 H, 6-H), 6.81 (d, *J* = 2.1 Hz, 1 H, 8-H), 5.88 (s, 2 H, 1′-H), 3.80 (s, 3 H, OCH_3_), 2.81 (s, 3 H, 1-CH_3_); ^13^C-NMR (124 MHz, CD_2_Cl_2_) *δ* = 161.3, 143.7, 140.7, 138.7, 135.9, 133.6, 132.9 (2 C), 130.9, 128.9, 127.8 (2 C). 126.5, 126.0, 124.2, 123.8, 122.5, 115.3, 112.3, 109.4, 93.4, 55.7, 53.2, 23.0; IR (KBr) $\tilde{\nu }$ (cm^−1^) = 3427, 3091, 1627, 1147; MS (EI) *m/z* (relative intensity, %) = 352 [M^+**•**^] (20), 141 (100); HRMS (EI) calcd. for C_24_H_20_N_2_O: 352.1576; found 352.1574; purity: 100% (λ = 210 nm), 100% (λ = 254 nm) (by HPLC).

*7-Methoxy-1-methyl-9-((E)-3-phenylallyl)-9H-pyrido[3,4-b]indole* (**AnnH22**). Prepared from harmine (100 mg, 0.472 mmol), potassium *tert*-butoxide (65 mg, 0.58 mmol), dry DMSO (10 mL) and *(E)*-3-phenylallyl bromide (509 mg, 2.58 mmol). The mixture was stirred at 80 °C for 3 h Beige solid (36 mg, 23%). m.p. 104–108 °C; ^1^H-NMR (400 MHz, CDCl_3_) *δ* = 8.30 (d, *J* = 5.3 Hz, 1 H, 3-H), 8.01 (d, *J* = 8.6 Hz, 1 H, 5-H), 7.78 (d, *J* = 5.2 Hz, 1 H, 4-H), 7.32–7.14 (m, 5 H, aromatic protons), 6.92 (dd, *J* = 8.6, 2.2 Hz, 1 H, 6-H), 6.85 (d, *J* = 2.0 Hz, 1 H, 8-H), 6.42 (dt, *J* = 16.0, 4.5 Hz, 1 H, 2‘-H), 6.21 (d, *J* = 16.0 Hz, 1 H, 3‘-H), 5.26 (dd, *J* = 4.4, 1.8 Hz, 2 H, 1‘-H), 3.92 (s, 3 H, OCH_3_), 3.03 (s, 3 H, 1-CH_3_); ^13^C-NMR (100 MHz, CDCl_3_) *δ* = 161.2, 143.4, 140.6, 138.1, 136.0, 135.5, 131.4, 129.7, 128.6 (2 C), 127.9 (2 C), 126.4, 124.7, 122.5, 115.1, 112.4, 109.3, 93.4, 55.7, 46.6, 22.7; IR (KBr) $\tilde{\nu }$ (cm^−1^) = 3362, 2926, 1624, 1169; MS (EI) *m/z* (relative intensity, %) = 328 [M^+**•**^] (36), 117 (100); HRMS (EI) calcd. for C_22_H_20_N_2_O: 328.1576; found 328.1574; purity: 92% (λ = 210 nm), 100% (λ = 254 nm) (by HPLC).

*7-Methoxy-1-methyl-9-(2-phenoxyethyl)-9H-pyrido[3,4-b]indole* (**AnnH40**). Prepared from harmine (150 mg, 0.709 mmol), sodium hydride (28 mg, 0.71 mmol), dry DMF (5.0 mL) and 2-bromoethyl phenyl ether (0.144 mg, 0.716 mmol). The mixture was stirred at 40 °C for 6 h. Beige solid (72 mg, 31%). m.p. 143–144 °C; ^1^H-NMR (500 MHz, CD_2_Cl_2_) *δ* = 8.24 (d, *J* = 5.2 Hz, 1 H, 3-H), 7.96 (d, *J* = 8.6 Hz, 1 H, 5-H), 7.72 (d, *J* = 5.2 Hz, 1 H, 4-H), 7.27–7.14 (m, 2 H, aromatic protons), 7.00 (d, *J* = 2.1 Hz, 1 H, 8-H), 6.92–6.87 (m, 2 H, 6-H, 4′-H), 6.78–6.76 (m, 2 H, aromatic protons), 4.91 (t, *J* = 5.7 Hz, 2 H, 1′-H), 4.35 (t, *J* = 5.7 Hz, 2 H, 2′-H), 3.92 (s, 3 H, OCH_3_), 3.05 (s, 3 H, 1-CH_3_); ^13^C-NMR (125 MHz, CD_2_Cl_2_) *δ* = 161.1, 158.4, 143.4, 141.1, 138.6, 135.8, 129.6 (2 C), 129.4, 122.3, 121.3, 115.3, 114.4 (2 C), 112.2, 109.4, 93.7, 67.0, 54.0, 44.3, 23.8; IR (KBr) $\tilde{\nu }$ (cm^−1^) = 3021, 2869, 1631, 1197; MS (EI) *m/z* (relative intensity, %) = 332 [M^+**•**^] (34), 225 (100), 182 (16); HRMS (EI) calcd. for C_21_H_20_N_2_O_2_: 332.1525; found 332.1528; purity: 100% (λ = 210 nm), 100% (λ = 254 nm) (by HPLC).

*7-Methoxy-1-methyl-9-(pyridin-4-yl-methyl)-9H-pyrido[3,4-b]indole* (**AnnH36**). Prepared from harmine (157 mg, 0.708 mmol), sodium hydride (41 mg, 0.71 mmol), dry DMF (5.0 mL) and 4-(chloromethyl)pyridine hydrochloride (0.125 mg, 0.708 mmol). The mixture was stirred at 40 °C for 7 h. Brown solid (20 mg, 9%). m.p. 143–146 °C; ^1^H-NMR (400 MHz, CD_2_Cl_2_) *δ* = 8.49 (dd, *J* = 4.5, 1.6 Hz, 2 H, 3′′-H, 5′′-H), 8.28 (d, *J* = 5.6 Hz, 1 H, 3- H), 8.11 (d, *J* = 8.7 Hz, 1 H, 5- H), 7.96 (d, *J* = 5.6 Hz, 1 H, 4-H), 7.00 (dd, *J* = 8.7, 2.1 Hz, 1 H, 6-H), 6.94 (d, *J* = 6.0 Hz, 2 H, 2′′-H, 6′′-H), 6.77 (d, *J* = 2.1 Hz, 1 H, 8-H), 5.78 (s, 2 H, 1′-H), 3.87 (s, 3 H, OCH_3_), 2.87 (s, 3 H, 1-CH_3_); ^13^C-NMR (100 MHz, CD_2_Cl_2_) *δ* = 162.8, 150.7 (2 C), 147.2, 145.0, 140.0, 136.2, 135.5, 131.7, 123.6, 121.1 (2 C), 114.9, 113.4, 111.2, 93.4, 56.1, 49.4, 21.1; IR (KBr) $\tilde{\nu }$ (cm^−1^) = 3425, 2922, 1620, 1166; MS (EI) *m/z* (relative intensity, %) = 303 [M^+**•**^] (100), 225 (26), 211 (95); HRMS (EI) calcd. for C_19_H_17_N_3_O: 303.1372; found 303.1371; purity: 93 (λ = 210 nm), 100% (λ = 254 nm) (by HPLC).

*[2-(7-Methoxy-1-methyl-9H-pyrido[3,4-b]indol-9-yl)-ethyl]-dimethylamine* (**AnnH16**). Prepared from harmine (103 mg, 0.490 mmol), sodium hydride (70 mg, 1.8 mmol), dry THF (5 mL) and *N*,*N*-dimethyl-2-chloroethylamine hydrochloride (107 mg, 0.750 mmol). Orange solid (91 mg, 66%). m.p. 88–92 °C; ^1^H-NMR (500 MHz, CDCl_3_) *δ* = 8.29 (d, *J* = 5.2 Hz, 1 H, 3-H), 7.97 (d, *J* = 7.1 Hz, 1 H, 5-H), 7.74 (d, *J* = 5.2 Hz, 1 H, 4-H), 6.91–6.89 (m, 2 H, 6-H, 8-H), 4.68–4.55 (m, 2 H, 1′-H), 3.95 (s, 3 H, OCH_3_), 3.06 (s, 3 H, 1-CH_3_), 2.76–2.63 (m, 2 H, 2′-H), 2.39 (s, 6 H, N(CH_3_)_2_); ^13^C-NMR (125 MHz, CDCl_3_) *δ* = 161.0, 143.1, 140.4, 138.2, 135.3, 129.5, 122.5, 115.2, 112.3, 109.1, 93.1, 58.6, 55.7, 46.0 (2 C), 43.5, 23.3; IR (KBr) $\tilde{\nu }$ (cm^−1^) = 3424, 2950, 1621, 1148; MS (EI) *m/z* (relative intensity, %) = 283 [M^+**•**^] (2), 58 (100); HRMS (EI) calcd. for C_17_H_21_N_3_O: 283.1685; found 283.1682; purity: 100% (λ = 210 nm), 100% (λ = 254 nm) (by HPLC).

*[3-(7-Methoxy-1-methyl-9H-pyrido[3,4-b]indol-9-yl)-N,N-dimethylpropan-1-amine* (**AnnH21**). Prepared from harmine (100 mg, 0.470 mmol), sodium hydride (65 mg, 1.6 mmol), dry THF (5 mL) and *N*,*N*-dimethyl-3-chloropropylamine hydrochloride (129 mg, 0.820 mmol). Orange solid (50 mg, 36%). m.p. 46–48 °C. The analytical data were in accordance with those published in ref. [33].

*4-[2-(7-Methoxy-1-methyl-9H-pyrido[3,4-b]indol-9-yl)-ethyl]morpholine* (**AnnH53**). Prepared from harmine (104 mg, 0.491 mmol), sodium hydride (68 mg, 1.7 mmol), dry THF (5 mL) and 4-(2-chloroethyl)morpholine hydrochloride (150 mg, 0.834 mmol). Beige solid (26 mg, 17%). m.p. 183 °C; ^1^H-NMR (400 MHz, CDCl_3_) *δ* = 8.29 (d, *J* = 5.4 Hz, 1 H, 3-H), 8.00 (d, *J* = 9.3 Hz, 1 H, 5-H), 7.79 (d, *J* = 5.4 Hz, 1 H, 4-H), 6.94–6.92 (m, 2 H, 6-H, 8-H), 4.71–4.58 (m, 2 H, 1′-H), 3.97 (s, 3 H, OCH_3_), 3.73–3.68 (m, 4 H, 5′′-H, 3′′-H), 3.12 (s, 3 H, 1-CH_3_), 2.83–2.69 (m, 2 H, 2′-H), 2.55–2.53 (m, 4 H, 2′′-H, 6′′-H); ^13^C-NMR (125 MHz, CDCl_3_) *δ* = 161.4, 143.6, 139.4, 136.9, 135.1, 130.2, 122.7, 115.0, 112.6, 109.5, 93.4, 66.8 (2 C), 58.0, 55.8, 54.2 (2 C), 43.0, 22.5; IR (KBr) $\tilde{\nu }$ (cm^−1^) = 3442, 2927, 1623, 1115; MS (ESI) *m/z =* 326 [M+ H], 235, 118; HRMS (EI) calcd. for C_19_H_23_N_3_O_2_: 325.1790; found 325.1789; purity: 90% (λ = 210 nm), 100% (λ = 254 nm) (by HPLC).

*7-Methoxy-1-methyl-9-[2-(pyrrolidin-1-yl)ethyl]-9H-pyrido[3,4-b]indole* (**AnnH55**). Harmine (150 mg, 0.706 mmol) and sodium hydride (137 mg, 3.43 mmol) were stirred in dry THF (7.5 mL) for 1 h. After addition of 1-(2-chloroethyl)pyrrolidine hydrochloride (204 mg, 1.20 mmol) the mixture was stirred for 23 h. Beige solid (100 mg, 46%). m.p. 99–101 °C; ^1^H-NMR (400 MHz, CDCl_3_) *δ* = 8.29 (d, *J* = 5.2 Hz, 1 H, 3-H), 7.97 (d, *J* = 8.6 Hz, 1 H, 5-H), 7.74 (d, *J* = 5.2 Hz, 1 H, 4-H), 6.96 (d, *J* = 1.9 Hz, 1 H, 8-H), 6.90 (dd, *J* = 8.6, 2.2 Hz, 1 H, 6-H), 4.76–4.61 (m, 2 H, 1′-H), 3.96 (s, 3 H, OCH_3_), 3.07 (s, 3 H, 1-CH_3_), 2.98–2.83 (m, 2 H, 2′-H), 2.70–2.66 (m, 4 H, pyrrolidin), 1.94–1.79 (m, 4 H, pyrrolidin); ^13^C-NMR (100 MHz, CDCl_3_) *δ* = 161.1, 143.1, 140.4, 138.3, 135.3, 129.5, 122.4, 115.2, 112.3, 109.3, 93.1, 55.8, 55.4, 54.6 (4 C), 44.1, 23.5; IR (KBr) $\tilde{\nu }$ (cm^−1^) = 3450, 2929, 1622, 1139; MS (ESI) *m/z =* 310 [M^+^+ H], 98; HRMS (EI) calcd. for C_19_H_23_N_3_O: 309.1841; found 309.1843; purity: 91% (λ = 210 nm), 100% (λ = 254 nm) (by HPLC).

*7-Methoxy-1-methyl-9-[2-(piperidin-1-yl)ethyl]-9H-pyrido[3,4-b]indole* (**AnnH57**). Harmine (106 mg, 0.501 mmol) and sodium hydride (93 mg, 0.23 mmol) were stirred in dry THF (5 mL) for 1 h. After addition of 1-(2-chloroethyl)piperidine hydrochloride (148 mg, 0.806 mmol) the mixture was stirred for 48 h. Light brown solid (75 mg, 33%). m.p. 108–110 °C; ^1^H-NMR (400 MHz, CDCl_3_) *δ* = 8.28 (d, *J* = 5.2 Hz, 1 H, 3-H), 7.96 (d, *J* = 8.6 Hz, 1 H, 5-H), 7.72 (d, *J* = 5.2 Hz, 1 H, 4-H), 6.93 (d, *J* = 2.0 Hz, 1 H, 8-H), 6.89 (dd, *J* = 8.6, 2.2 Hz, 1 H, 6-H), 4.71–4.56 (m, 2 H, 1′-H), 3.94 (s, 3 H, OCH_3_), 3.05 (s, 3 H, 1-CH_3_), 2.77–2.63 (m, 2 H, 2′-H), 2.55–2.50 (m, 4 H, 1′′-H, 5′′-H), 1.62–1.59 (m, 4 H, 2′′-H, 4′′-H), 1.48–1.42 (m, 2 H, 3′′-H); ^13^C-NMR (100 MHz, CDCl_3_) *δ* = 160.9, 143.1, 140.6, 138.3, 135.4, 129.4, 122.4, 115.2, 112.3, 109.0, 93.2, 58.3, 55.7, 55.3 (2 C), 42.9, 25.9 (2 C), 24.1, 23.5; IR (KBr) $\tilde{\nu }$ (cm^−1^) = 3444, 2932, 1621, 1112; MS (ESI) *m/z =* 324 [M^+^+ H], 163, 112, 102; HRMS (EI) calcd. for C_20_H_25_N_3_O: 323.1998; found 323.1999; purity: 100% (λ = 210 nm), 100% (λ = 254 nm) (by HPLC).

*Ethyl 2-(7-methoxy-1-methyl-9H-pyrido[3,4-b]indol-9-yl)acetate* (**AnnH12**). The preparation of **AnnH12** has been reported in a patent [35] without presenting spectroscopic data. ^1^H-NMR (400 MHz, CDCl_3_) *δ* = 8.30 (d, *J* = 5.2 Hz, 1 H, 3-H), 7.98 (d, *J* = 8.6 Hz, 1 H, 5-H), 7.74 (d, *J* = 5.3 Hz, 1 H, 4-H), 6.91 (dd, *J* = 2.2, 8.6 Hz, 1 H, 6-H), 6.76 (d, *J* = 2.2, Hz, 1 H, 8-H), 5.20 (s, 2 H, 1′-H), 4.27–4.20 (m, 2 H, O-CH_2_), 3.93 (s, 3 H, OCH_3_), 2.94 (s, 3 H, 1-CH_3_), 1.25 (m, 3 H, CH_2_-CH_3_); ^13^C-NMR (100 MHz, CDCl_3_) *δ* = 168.6, 161.1, 143.4, 140.5, 139.0, 135.7, 129.8, 122.6, 115.4, 112.5, 109.4, 93.0, 62.0, 55.7, 46.8, 23.0, 14.1; IR (KBr) $\tilde{\nu }$ (cm^−1^) = 3461, 2872, 1736, 1622, 1252; MS (EI) *m/z* (relative intensity, %) *=* 298 [M^+**•**^] (50), 225 (100), 182 (24); HRMS (EI) calcd. for C_17_H_18_N_2_O_3_: 298.1318; found 298.1311.

*Methyl 2-(7-methoxy-1-methyl-9H-pyrido[3,4-b]indol-9-yl)acetate* (**AnnH26**). Prepared from harmine (105 mg, 0.490 mmol), sodium hydride (20 mg, 0.49 mmol), dry DMF (4.0 mL) and methyl bromoacetate (0.050 mL, 0.53 mmol). The mixture was stirred at 40 °C for 4 h. White solid (16 mg, 11%). m.p. 121–125 °C. ^1^H-NMR (500 MHz, CDCl_3_) *δ* = 8.31 (d, *J* = 5.2 Hz, 1 H, 3-H), 7.98 (d, *J* = 8.6 Hz, 1 H, 5-H), 7.74 (d, *J* = 5.2 Hz, 1 H, 4-H), 6.91 (dd, *J* = 8.6, 2.0 Hz, 1 H, 6-H), 6.75 (d, *J* = 2.1 Hz, 1 H, 8-H), 5.21 (s, 2 H, 1′-H), 3.93 (s, 3 H, OCH_3_), 3.77 (s, 3 H, COOCH_3_), 2.93 (s, 3 H, 1-CH_3_); ^13^C-NMR (100 MHz, CDCl_3_) *δ* = 169.4, 161.2, 143.3, 140.5, 139.0, 135.6, 129.8, 122.6, 115.4, 112.5, 109.4, 93.0, 55.7, 52.9, 46.7, 23.0; IR (KBr) $\tilde{\nu }$ (cm^−1^) = 3446, 2998, 1735, 1624, 1174; MS (EI) *m/z* (relative intensity, %) *=* 284 [M^+**•**^] (37), 225 (100), 182 (26); HRMS (EI) calcd. for C_16_H_16_N_2_O_3_: 284.1161; found 284.1162; purity: 100% (λ = 210 nm), 100% (λ = 254 nm) (by HPLC).

*Isopropyl 2-(7-methoxy-1-methyl-9H-pyrido[3,4-b]indol-9-yl)acetate***(AnnH27)**. Prepared from harmine (150 mg, 0.706 mmol), potassium *tert*-butoxide (144 mg, 1.28 mmol), dry DMSO (13 mL) and isopropyl bromoacetate (1.0 mL, 7.7 mmol). The mixture was stirred at 80 °C for 3 h. Beige solid (18 mg, 10%). m.p. 78–82 °C; ^1^H-NMR (400 MHz, CDCl_3_) *δ* = 8.30 (d, *J* = 5.3 Hz, 1 H, 3-H), 7.97 (d, *J* = 8.6 Hz, 1 H, 5-H), 7.74 (d, *J* = 5.2 Hz, 1 H, 4-H), 6.91 (dd, *J* = 8.6, 2.1 Hz, 1 H, 6-H), 6.76 (d, *J* = 2.0 Hz, 1 H, 8-H), 5.15 (s, 2 H, 1′-H), 5.12–5.10 (m, 1 H, OCH), 3.96 (s, 3 H, OCH_3_), 2.93 (s, 3 H, 1-CH_3_), 1.24 (s, 6 H, CH(CH_3_)_2_); ^13^C-NMR (100 MHz, CDCl_3_) *δ* = 168.3, 161.7, 143.5, 140.5, 138.8, 135.7, 129.8, 122.5, 115.3, 112.4, 109.3, 93.0, 69.8, 55.6, 47.0, 22.9, 21.7 (2 C); IR (KBr) $\tilde{\nu }$ (cm^−1^) = 3426, 2980, 1729, 1632, 1175; MS (EI) *m/z* (relative intensity, %) = 312 [M^+**•**^] (28), 270 (8), 225 (100), 182 (16); HRMS (EI) calcd. for C_18_H_20_N_2_O_3_: 312.1474; found 312.1474; purity: 100% (λ = 210 nm), 100% (λ = 254 nm) (by HPLC).

*Ethyl 3-(7-Methoxy-1-methyl-9H-pyrido[3,4-b]indol-9-yl)propanoate* (**AnnH32**). To harmine (105 mg, 0.494 mmol) in dry DMF (3.0 mL) was added sodium hydride (60% in mineral oil, 49 mg, 1.2 mmol). The mixture was stirred at room temperature for 1 h. Ethyl 3-bromopropionate (0.070 mL, 0.54 mmol) was added and the mixture was stirred for 18 h at room temperature. Then, additional sodium hydride (60% in mineral oil, 26 mg, 0.65 mmol) and ethyl 3-bromopropionate (0.070 mL, 0.54 mmol) were added and the mixture was stirred for 1 h. The reaction mixture was treated with DCM (20 mL) and washed with water (20 mL) and brine (20 mL). The organic layer was dried over magnesium sulfate and evaporated to dryness. The residue was purified by coulmn chromatography (silica gel, iso-hexane/ethyl acetate/ethanol 2:2:1) to afford **AnnH32** as a white solid (18 mg, 12%). m.p. 95–96 °C; ^1^H-NMR (500 MHz, CDCl_3_) *δ* = 8.30 (d, *J =* 5.2 Hz, 1 H, 3-H), 7.97 (d, *J* = 8.6 Hz, 1 H, 5- H), 7.74 (d, *J* = 5.2 Hz, 1 H, 4-H), 6.93 (d, *J* = 2.0 Hz, 1 H, 8-H), 6.90 (dd, *J* = 8.6, 2.1 Hz, 1 H, 6-H), 4.90–4.77 (m, 2 H, 1′-H), 4.12 (q, *J* = 7.2 Hz, 2 H, O-CH_2_), 3.96 (s, 3 H, OCH_3_), 3.04 (s, 3 H, 1-CH_3_), 2.86–2.73 (m, 2 H, 2′-H), 1.24–1.13 (m, 3 H, CH_2_-CH_3_); ^13^C-NMR (100 MHz, CDCl_3_) *δ* = 170.9, 161.0, 142.7, 140.5, 138.6, 135.0, 129.8, 122.5, 115.3, 112.3, 109.3, 93.2, 61.1, 55.7, 40.5, 35.0, 23.3, 14.0; IR (KBr) $\tilde{\nu }$ (cm^−1^) = 3445, 2968, 1728, 1622, 1196; MS (EI) *m/z* (relative intensity, %) = 312 [M^+**•**^] (48), 225 (100), 182 (15); HRMS (EI) calcd. for C_18_H_20_N_2_O_3_: 312.1474; found 312.1474; purity: 100% (λ = 210 nm), 100% (λ = 210 nm) (by HPLC).

*2-(7-Methoxy-1-methyl-9H-pyrido[3,4-b]indol-9-yl)acetic acid* (**AnnH25**). Ethyl-2-(7-methoxy-1-methyl-9*H*-pyrido[3,4-*b*]indol-9-yl)acetate (**AnnH12**) (107 mg, 0.360 mmol) was dissolved in methanol (3.45 mL). After addition of water (3.45 mL) and aqueos sodium hydroxide (1 M, 0.38 mL, 0.38 mmol) the mixture was stirred at room temperature for 4 h. The solvent was removed under reduced pressure and water (5 mL) was added. The pH was adjusted to 5–6 with 2 M HCl. The formed precipitate was collected by filtration, washed with water and dried in vacuo to afford **AnnH25** as a white solid (73 mg, 75%). m.p. 276–279 °C; ^1^H-NMR (400 MHz, DMSO-*d_6_*) *δ* = 9.90 (s, 1 H, OH), 8.18 (d, *J* = 5.2 Hz, 1 H, 3-H), 8.10 (d, *J* = 8.6 Hz, 1 H, 5-H), 7.91 (d, *J* = 5.3 Hz, 1 H, 4-H), 7.29 (d, *J* = 2.0 Hz, 1 H, 8-H), 6.88 (dd, *J* = 8.6, 2.0 Hz, 1 H, 6-H), 5.41 (s, 2 H, 1′-H), 3.90 (s, 3 H, OCH_3_), 2.85 (s, 3 H, 1-CH_3_); ^13^C-NMR (100 MHz, DMSO-*d_6_*) *δ* = 171.1, 160.6, 143.4, 140.4, 137.6, 135.0, 128.6, 122.3, 113.9, 112.3, 109.5, 93.5, 55.5, 46.0, 22.2; IR (KBr) $\tilde{\nu }$ (cm^−1^) = 3506, 2928, 1628, 1134; MS (EI) *m/z* (relative intensity, %) = 270 [M^+**•**^] (72), 225 (100), 182 (30), 142 (20), 83 (57); HRMS (EI) calcd. for C_15_H_14_N_2_O_3_: 270.1005; found 270.0970; purity: 100% (λ = 210 nm), 99% (λ = 210 nm) (by HPLC).

*2-(7-Methoxy-1-methyl-9H-pyrido[3,4-b]indol-9-yl)-N-acetamide* (**AnnH28**). To harmine (103 mg, 0.486 mmol) in dry DMF (3.0 mL) was added sodium hydride (22 mg, 0.54 mmol). The mixture was stirred at 40 °C for 30 min. Then, it was cooled to room temperature and 2-bromoacetamide (67 mg, 0.49 mmol) was added. The mixture was stirred at room temperature for 24 h, then poured into ice water (25 mL) and extracted with ethyl acetate (3 × 20 mL). The combined organic layers were washed with water (2 × 60 mL), dried over magnesium sulfate and evaporated to dryness. The residue was purified by column chromatography (silica gel, iso-hexane/ethyl acetate/ethanol/triethylamine 4:4:3 +1%) to afford **AnnH28** as a beige solid (18 mg, 14%). m.p. 291–297 °C; ^1^H-NMR (400 MHz, DMSO-*d_6_*) *δ* = 8.21 (d, *J* = 5.4 Hz, 1 H, 3-H), 8.16 (d, *J* = 8.6 Hz, 1 H, 5-H), 8.04 (d, *J* = 5.4 Hz, 1 H, 4-H), 7.80 (s, 1 H, NH_2_), 7.42 (s, 1 H, NH_2_), 7.21 (d, *J* = 2.2 Hz, 1 H, 8-H), 6.92 (dd, *J* = 8.5, 2.0 Hz, 1 H, 6-H), 5.23 (s, 2 H, 1′-H), 3.90 (s, 3 H, OCH_3_), 2.91 (s, 3 H, 1-CH_3_); ^13^C-NMR (100 MHz, DMSO-*d_6_*) *δ* = 170.0, 161.2, 144.3, 140.0, 136.0, 135.2, 129.5, 122.7, 114.0, 112.7, 110.0, 93.6, 55.7, 47.1, 21.4; IR (KBr) $\tilde{\nu }$ (cm^−1^) = 3423, 3013, 1683, 1196; MS (EI) *m/z* (relative intensity, %) = 269 [M^+**•**^] (32), 225 (100), 182 (25); HRMS (EI) calcd. for C_15_H_15_N_3_O_2_: 269.1164; found 264.1164; purity: 100% (λ = 210 nm), 100% (λ = 254 nm) (by HPLC).

*2-(7-Methoxy-1-methyl-9H-pyrido[3,4-b]indol-9-yl)-N-methylacetamide* (**AnnH30**). Prepared from harmine (150 mg, 0.708 mmol), sodium hydride (76 mg, 1.9 mmol), dry DMF (5.0 mL) and 2-chloro-*N*-methylacetamide (81 mg, 0.75 mmol). The mixture was stirred at 40 °C for 6 h and over night at room temperature. Then, additional 2-chloro-*N*-methylacetamide (81 mg, 0.75 mmol) was added and the mixture was stirred at 40 °C for 5 h, then poured into ice water (25 mL) and extracted with ethyl acetate (3 × 20 mL). The combined organic layers were washed with water (2 × 60 mL), dried over magnesium sulfate, and evaporated to dryness. The residue was purified by column chromatography (silica gel, iso-hexane/ethyl acetate/ethanol/triethylamine 2:2:1 + 1%) to afford **AnnH30** as a beige solid (65 mg, 33%). m.p. 265–272 °C; ^1^H-NMR (400 MHz, DMSO-*d_6_*) *δ* = 8.21 (d, *J* = 4.6 Hz, 1 H, NH), 8.17 (d, *J =* 5.2 Hz, 1 H, 3-H), 8.10 (d, *J* = 8.6 Hz, 1 H, 5-H), 7.90 (d, *J* = 5.2 Hz, 1 H, 4 H), 7.17 (d, *J* = 2.0 Hz, 1 H, 8-H), 6.88 (dd, *J* = 8.6, 2.1 Hz, 1 H, 6-H), 5.20 (s, 2 H, 1′-H), 3.88 (s, 3 H, OCH_3_), 2.84 (s, 3 H, 1-CH_3_), 2.64 (d, *J* = 4.6 Hz, 3 H, N-CH_3_); ^13^C-NMR (100 MHz, DMSO-*d_6_*) *δ* = 168.6, 160.6, 143.6, 140.9, 137.7, 135.4, 128.7, 122.4, 114.3, 112.3, 109.4, 93.6, 55.7, 40.1, 25.7, 22,5; IR (KBr) $\tilde{\nu }$ (cm^−1^) = 3424, 2925, 1656, 1193; MS (EI) *m/z* (relative intensity, %) = 283 [M^+**•**^] (28), 225 (100), 182 (24); HRMS (EI) calcd. for C_16_H_17_N_3_O_2_: 283.1321; found 283.1322; purity: 100%(λ = 210 nm), 92% (λ = 254 nm) (by HPLC).

*2-(7-Methoxy-1-methyl-9H-pyrido[3,4-b]indol-9-yl)-N,N-dimethylacetamide* (**AnnH34**). The preparation of **AnnH34** has been reported in in a patent [35] without presenting spectroscopic data. ^1^H-NMR (500 MHz, CDCl_3_) *δ* = 8.27 (d, *J* = 5.2 Hz, 1 H, 3-H), 7.96 (d, *J* = 8.6 Hz, 1 H, 5-H), 7.72 (d, *J* = 5.2 Hz, 1 H, 4-H), 6.87 (dd, *J* = 8.6, 2.1 Hz, 1 H, 6-H), 6.67 (d, *J* = 2.0 Hz, 1 H, 8-H), 5.19 (s, 2 H, 1′-H), 3.90 (s, 3 H, OCH_3_), 3.19 (s, 3 H, 1-CH_3_), 3.03 (s, 3 H, N-CH_3_), 2.87 (s, 3 H, N-CH_3_); ^13^C-NMR (100 MHz, CDCl_3_) *δ* = 167.2, 161.0, 143.8, 140.3, 138.6, 136.1, 129.7, 122.5, 115.4, 112.5, 108.9, 93.2, 55.7, 46.5, 36.4, 36.1, 22.9; IR (KBr) $\tilde{\nu }$ (cm^−1^) = 3496, 2959, 1650, 1177; MS (EI) *m/z* (relative intensity, %) = 297 [M^+**•**^] (23), 225 (100), 182 (27); HRMS (EI) calcd. for C_17_H_19_N_3_O_2_: 297.1477; found 297.147; purity: 100%, 100% (λ = 210 nm) (by HPLC).

*2-(7-Methoxy-1-methyl-9H-pyrido[3,4-b]indol-9-yl)-acetonitrile* (**AnnH31**). The preparation of **AnnH31** has been reported previously in a patent [35] without spectroscopic data. ^1^H-NMR (500 MHz, CDCl_3_) *δ* = 8.36 (d, *J* = 5.2 Hz, 1 H, 3-H), 7.97 (d, *J* = 8.6 Hz, 1 H, 5- H), 7.72 (d, *J* = 5.2 Hz, 1 H, 4-H), 6.96 (dd, *J* = 8.6, 2.0 Hz, 1 H, 6-H), 6.86 (d, *J* = 1.9 Hz, 1 H, 8-H), 5.35 (s, 2 H, 1′-H), 3.96 (s, 3 H, OCH_3_), 3.08 (s, 3 H, 1-CH_3_); ^13^C-NMR (125 MHz, CDCl_3_) *δ* = 161.5, 142.4, 140.4, 140,3, 134.8, 130.5, 122.9, 115.7, 114.7, 112.6, 110.3, 93.2, 55.8, 33.6, 23.0; IR (KBr) $\tilde{\nu }$ (cm^−1^) = 3441, 2924, 2208, 1629; MS (EI) *m/z* (relative intensity, %) = 251 [M^+**•**^] (100), 225 (10), 211 (68), 168 (23); HRMS (EI) calcd. for C_15_H_13_N_3_O: 251.1059; found 251.1059.

*3-(7-Methoxy-1-methyl-9H-pyrido[3,4-b]indol-9-yl)propanenitrile* (**AnnH42**). A suspension of harmine (102 mg, 0.482 mmol) in acrylonitrile (2.0 mL, 30 mmol) was stirred in an ice-bath for 15 min. Then, benzyltriethylammonium hydroxide (40% in methanol, 3.5 µL, 0.0062 mmol) was added dropwise and the mixture was stirred at room temperature for 3 h and at 70 °C for 1h. The crude product that precipitated in the flask upon cooling was recrystallized from acetonitrile to afford **AnnH42** as white solid (35 mg, 27%). m.p. 190 °C; ^1^H-NMR (400 MHz, CDCl_3_) *δ* = 8.34 (d, *J* = 5.2 Hz, 1 H, 3-H), 7.99 (d, *J =* 8.6 Hz, 1 H, 5-H), 7.74 (d, *J* = 5.2 Hz, 1 H, 4-H), 6.94 (dd, *J* = 8.6, 2.1 Hz, 1 H, 6-H), 6.88 (d, *J* = 2.0 Hz, 1 H, 8-H), 4.92–4.79 (m, 2 H, 1′-H), 3.97 (s, 3 H, OCH_3_), 3.03 (s, 3 H, 1-CH_3_), 2.89–2.76 (m, 2 H, 2′-H); ^13^C-NMR (400 MHz, CDCl_3_) *δ* = 161.3, 142.2, 140.1, 139.5, 134.7, 130.1, 122.8, 116.7, 115.7, 112.5, 109.6, 93.2, 55.8, 40.6, 23.53, 18.4; IR (KBr) $\tilde{\nu }$ (cm^−1^) = 3442, 2990, 2345, 1627, 1173; MS (ESI) *m/z =* 266 [M^+^+ H], 225, 231, 150; HRMS (EI) calcd. for C_16_H_15_N_3_O: 265.1215; found 265.1216; purity: 100% (λ = 210 nm), 100% (λ = 254 nm) (by HPLC).

*(±)-2-(7-Methoxy-1-methyl-9H-pyrido[3,4-b]indol-9-yl)propanenitrile* (**AnnH43**). Prepared from harmine (150 mg, 0.706 mmol), sodium hydride (40 mg, 1.0 mmol), dry DMF (5.0 mL) and (±)-2-bromopropionitrile (0.060 mL, 0.69 mmol). The mixture was stirred at 40 °C for 7 h. Beige solid (39 mg, 21%). m.p. 132–134 °C; ^1^H-NMR (500 MHz, CDCl_3_) *δ* = 8.35 (d, *J* = 5.2 Hz, 1 H, 3-H), 8.00 (d, *J* = 8.6 Hz, 1 H, 5-H), 7.74 (d, *J* = 5.2 Hz, 1 H, 4-H), 7.26 (d, *J* = 2.1 Hz, 1 H, 8-H), 6.98 (dd, *J* = 8.6, 2.1 Hz, 1 H, 6-H), 6.25 (q, *J* = 7.2 Hz, 1 H, CH(CH_3_)), 3.98 (s, 3 H, OCH_3_), 3.01 (s, 3 H, 1-CH_3_), 1.95 (d, *J* = 7.3 Hz, 3 H, CH(CH_3_)); ^13^C-NMR (100 MHz, CDCl_3_) *δ* = 161.0, 140.7, 139.8, 139.6, 134.6, 130.4, 122.7, 117.2, 116.4, 112.6, 110.2, 95.6, 55.8, 41.9, 23.9, 19.3; IR (KBr) $\tilde{\nu }$ (cm^−1^) = 3428, 2918, 2369, 1628, 1211; MS (EI) *m/z* (relative intensity, %) = 265 [M^+**•**^] (78), 211 (100), 197 (20), 169 (38); HRMS (EI) calcd. for C_16_H_15_N_3_O: 265.1215; found 265.1209; purity: 100% (λ = 210 nm), 100% (λ = 254 nm) (by HPLC).

*7,8-Dichloro-2,9-dihydro-1H-pydrido[3,4-b]indol-1-one* (**AnnH65**). To a solution of 6,7-dichloro-2,3,4,9-tetrahydro-1*H*-pyrido[3,4-*b*]indol-1-one (**1**) [36] (1.94 g, 7.61 mmol) in 120 mL of dry THF was added 2,3-dichloro-5,6-dicyano-1,4-benzoquinone (3.46 g, 15.0 mmol) under a N_2_ atmosphere. The mixture was stirred under reflux for 15 h and then cooled to room temperature. After addition of ethyl acetate (55 mL), the reaction mixture was washed with 1 N sodium hydroxide solution (4 × 100 mL). The combined aqueous layers were extracted with ethyl acetate (2 × 100 mL). The combined organic layers were dried over sodium sulfate and evaporated to dryness. The beige residue was washed with hot ethyl acetate, methanol and DCM to afford **AnnH65** as a beige solid (1.27 g, 67%). m.p. 200 °C; ^1^H-NMR (500 MHz, DMSO-*d_6_*) *δ* = 12.52 (s, 1 H, 9-H), 11.58 (s, 1 H, 2-H), 8.07 (d, *J* = 8.2 Hz, 1 H, 5-H), 7.40 (d, *J* = 8.2 Hz, 1 H, 6-H), 7.19 (d, *J* = 6.4 Hz, 1 H, 3-H), 7.03 (d, *J* = 6.4 Hz, 1 H, 4-H); ^13^C-NMR (100 MHz, DMSO-*d_6_*) *δ =* 155.3, 137.0, 129.3, 128.7, 126.0, 125.1, 122.4, 121.2, 121.1, 115.2, 99.2; IR (KBr) $\tilde{\nu }$ (cm^−1^) = 3434, 3114, 1739, 1644, 1161; MS (EI) *m/z* (relative intensity, %) *=* 256 (6), 254 (66), 252 [M^+**•**^] (100); HRMS (EI) calcd. for C_11_H_6_N_2_Cl_2_O: 251.9857; found 251.9847; purity: 100% (λ = 210 nm), 100% (λ = 254 nm) (by HPLC).

*1-Bromo-7,8-dichloro-9H-pyrido[3,4-b]indole***(AnnH52**). To a solution of 7,8-dichloro-2,9-dihydro-1*H*-pyrido[3,4-*b*]indol-1-one (**AnnH65**) (1.27 g, 5.04 mmol) in anisole (20 mL) was added phosphorus oxybromide (10.0 g, 35.0 mmol). The mixture was stirred at 120 °C for 4 h, then cooled to room temperature, and saturated aqueuos sodium carbonate solution (130 mL) was added. The mixture was extracted with ethyl acetate (4 × 200 mL). The combined organic layers were dried over sodium sulfate and evaporated to dryness. The residue was purified by column chromatography (silica gel, iso-hexane/ethyl acetate 1:1) to afford **AnnH52** as a beige solid (1.27 g, 80%). m.p. 200 °C; ^1^H-NMR (500 MHz, DMSO-*d_6_*) *δ* = 12.15 (s, 1 H, NH), 8.29 (d, *J* = 8.4 Hz, 1 H, 5-H), 8.26 (d, *J* = 5.2 Hz, 1 H, 4-H), 8.22 (d, *J* = 5.2 Hz, 1 H, 3-H), 7.54 (d, *J* = 8.4 Hz, 1 H, 6-H); ^13^C-NMR (125 MHz, DMSO-*d_6_*) *δ* = 140.9, 139.2, 136.2, 131.8, 130.2, 125.0, 122.6, 122.4, 122.3, 115.9, 115.8; IR (KBr): $\tilde{\nu }$ (cm^−1^) = 3423, 1624, 798; MS (ESI): *m/z =* 319, 317, 315 [M^+^+H], 280, 248, 113; HRMS (EI) calcd. for C_11_H_5_N_2_Cl_2_Br: 313.9013; found 313.9005; purity: 96% (λ = 254 nm), 100% (λ = 254 nm) (by HPLC).

*7,8-Dichloro-1-methyl-9H-pyrido[3,4-b]indole* (**AnnH63**). 1-Bromo-7,8-dichloro-9*H*-[3,4-*b*] indole (**AnnH52**) (69 mg, 0.22 mmol) and tetrakis(triphenylphosphine)palladium(0) (15 mg, 0.013 mmol) were dissolved under a N_2_ atmosphere in 5 mL of dioxane. To this reaction trimethylaluminium (0.12 mL of 2 M solution in hexane, 0.24 mmol) was added. The mixture was stirred at reflux for 3 h and then cooled to room temperature. After the addition of water (25 mL) the mixture was extracted with ethyl acetate (3 × 50 mL). The combined organic layers were dried over magnesium sulfate and evaporated to dryness. The white solid was purified by precipitation with using of DCM and n-hexane to afford **AnnH63** as a white solid (51 mg, 92%). m.p. 226–227 °C; ^1^H-NMR (400 MHz, CDCl_3_) *δ* = 8.65 (s, 9-H), 8.41 (d, *J* = 5.4 Hz, 1 H, 3-H), 7.91 (d, *J* = 8.4 Hz, 1 H, 5-H), 7.76 (d, *J* = 5.4 Hz, 1 H, 4-H), 7.36 (d, *J* = 8.4 Hz, 1 H, 6-H), 2.87 (s, 3 H, 1-CH_3_); ^13^C-NMR (100 MHz, CDCl_3_) *δ* = 142.6, 139.7, 138.2, 134.6, 131.5, 128.4, 122.1, 121.7, 120.6, 115.6, 113.0, 20.4; IR (KBr) $\tilde{\nu }$ (cm^−1^) = 3425, 2926, 1618, 942; MS (ESI) *m/z =* 255, 253, 251 [M^+^+H], 102; HRMS (ESI) calcd. for C_12_H_8_Cl_2_N_2_+ H^+^: 251.0143; found 251.0136; purity: 100% (λ = 210 nm), 100% (λ = 254 nm) (by HPLC).

*Ethyl 2-(7,8-Dichloro-1-methyl-9H-pyrido[3,4-b]-9-yl)acetate* (**AnnH66**). Prepared from **AnnH63** (81 mg, 0.32 mmol), sodium hydride (17 mg, 0.41 mmol), dry DMF (3.0 mL) and ethyl bromoacetate (0.040 mL, 0.36 mmol). The mixture was stirred at 40 °C for 4 h. Beige solid (22 mg, 21%). m.p. 110–111 °C; ^1^H-NMR (400 MHz, CDCl_3_) *δ* = 8.40 (d, *J* = 5.2 Hz, 1 H, 3-H), 7.94 (d, *J* = 8.3 Hz, 1 H, 5-H), 7.78 (d, *J* = 5.3 Hz, 1 H, 4-H), 7.41 (d, *J* = 8.3 Hz, 1 H, 6-H), 6.08–5.27 (m, 2 H, 1′-H), 4.29 (q, *J* = 7.1 Hz, 2 H, O-CH_2_), 2.96 (s, 3 H, 1-CH_3_), 1.30–1.26 (m, 3 H, CH_2_-CH_3_); ^13^C-NMR (100 MHz, CDCl_3_) *δ* = 169.3, 142.0, 139.9, 138.5, 137.4, 133.7, 129.0, 123.4, 122.8, 120.2, 115.8, 112.7, 62.0, 48.5, 23.7, 14.2; IR (KBr) $\tilde{\nu }$ (cm^−1^) = 3430, 3059, 1743, 1655, 1165; MS (APCI) *m/z =* 341, 339, 337 [M^+^+ H], 251; HRMS (ESI) calcd. for C_16_H_14_N_2_O_2_Cl_2_ + H^+^: 337.0510; found 337.0508; purity: 92% (λ = 210 nm), 97% (λ = 254 nm) (by HPLC).

*2-(7,8-Dichloro-1-methyl-9H-pyrido[3,4-b]indol-9-yl)acetonitrile* (**AnnH69**). Prepared from **AnnnH63** (102 mg, 0.404 mmol), sodium hydride (18 mg, 0.45 mmol), dry DMF (2.5 mL) and bromoacetonitrile (0.030 mL, 0.44 mmol). The mixture was stirred at 40 °C for 4 h and overnight at room temperature. Then, additional bromoacetonitrile (0.030 mL, 0.44 mmol) was added and the mixture was stirred at 40 °C for 2 h. Brown solid (17 mg, 15%). m.p. 180–181 °C; ^1^H-NMR (500 MHz, CDCl_3_) *δ* = 8.36 (d, *J* = 5.3 Hz, 1 H, 3-H), 7.87 (d, *J* = 8.3 Hz, 1 H, 5-H), 7.73 (d, *J* = 5.3 Hz, 1 H, 4-H), 7.43 (d, *J* = 8.3 Hz, 1 H, 6-H), 5.75 (s, 2 H, 1′-H), 3.04 (s, 3 H, 1-CH_3_); ^13^C-NMR (100 MHz, CDCl_3_) *δ* = 142.5, 141.2, 138.5, 137.3, 134.6, 130.5, 124.5, 124.2, 120.6, 116.6, 115.5, 113.1, 35.6, 23.0; IR (KBr) $\tilde{\nu }$ (cm^−1^) = 3433, 2983, 2364, 1654, 1066; MS (APCI) *m/z =* 294, 292, 290 [M^+^+ H], 136; HRMS (ESI) calcd. for C_14_H_9_N_3_Cl_2_ + H^+^: 290.0252; found 290.0249; purity: 97% (λ = 210 nm), 100% (λ = 254 nm) (by HPLC).

*7,8-Dichloro-1-methyl-9-(prop-2-yn-1-yl)-9H-pyrido[3,4-b]indole* (**AnnH67**). Prepared from **AnnnH63** (79 mg, 0.31 mmol), sodium hydride (15 mg, 0.37 mmol), dry DMF (2.5 mL) and propargyl bromide (80 wt % in toluene, 0.040 mL, 0.35 mmol). The mixture was stirred at 40 °C for 3 h. Beige solid (41 mg, 45%). m.p. 174–175 °C; ^1^H-NMR (500 MHz, CDCl_3_) *δ* = 8.38 (d, *J* = 5.2 Hz, 1 H, 3-H), 7.88 (d, *J* = 8.3 Hz, 1 H, 5-H), 7.78 (d, *J* = 5.3 Hz, 1 H, 4-H), 7.38 (d, *J* = 8.3 Hz, 1 H, 6-H), 5.67 (d, *J* = 2.2 Hz, 2 H, 1′-H), 3.13 (s, 3 H, 1-CH_3_), 2.38 (t, *J* = 2.4 Hz, 1 H, 3′-H); ^13^C-NMR (100 MHz, CDCl_3_) *δ* = 142.9, 140.1, 138.1, 137.1, 133.8, 129.3, 123.7, 122.9, 120.0, 116.3, 112.5, 79.4, 74.4, 36.7, 23.6; IR (KBr) $\tilde{\nu }$ (cm^−1^) = 3426, 3160, 2107, 1613, 1062; MS (APCI) *m/z =* 293, 291, 289 [M^+^+ H], 250; HRMS (ESI) calcd. for C_15_H_10_N_2_Cl_2_ + H^+^: 289.0299; found 289.0296; purity: 100% (λ = 210 nm), 99% (λ = 254 nm) (by HPLC).

*3-(7,8-Dichloro-1-methyl-9H-pyrido[3,4-b]-9-yl)-N,N-dimethylpropan-1-amine* (**AnnH71**). Prepared from 7,8-dichloro-1-methyl-9*H*-pyrido[3,4-*b*]indole (**AnnH63**) (92 mg, 0.37 mmol), sodium hydride (57 mg, 1.4 mmol), dry THF (5 mL) and *N,N*-dimethyl-3-chloropropylamine hydrochloride (133 mg, 0.843 mmol). Beige solid (35 mg, 28%). m.p. 126–128 °C; ^1^H-NMR (500 MHz, CDCl_3_) *δ* = 8.35 (d, *J* = 5.2 Hz, 1 H, 3-H), 7.90 (d, *J* = 8.3 Hz, 1 H, 5-H), 7.74 (d, *J* = 5.3 Hz, 1 H, 4-H), 7.36 (d, *J* = 8.3 Hz, 1 H, 6-H), 5.00–4.90 (m, 2 H, 1′-H), 3.06 (s, 3 H, 1-CH_3_), 2.19 (t, *J* = 7.0 Hz, 2 H, 3′-H), 2.15 (s, 6 H, N(CH_3_)_2_), 1.96–1.81 (m, 2 H, 2′-H); ^13^C-NMR (100 MHz, CDCl_3_) *δ* = 142.7, 139.2, 138.1, 137.1, 133.5, 128.7, 123.6, 122.2, 120.0, 116.0, 112.4, 56.2, 45.4 (2 C), 44.3, 30.0, 24.3; IR (KBr) $\tilde{\nu }$ (cm^−1^) = 3426, 2932, 1628, 1051; MS (APCI) *m/z =* 340, 338, 336 [M^+^+ H], 149; HRMS (ESI) calcd. for C_17_H_19_N_3_Cl_2_ + H^+^: 336.1034; found 336.1031; purity: 100% (λ = 210 nm), 100% (λ = 254 nm) (by HPLC).

*2-(1-Bromo-7,8-dichloro-9H-pyrido[3,4-b]indol-9-yl)-acetonitrile* (**AnnH79**). Prepared from **AnnnH52** (102 mg, 0.404 mmol), sodium hydride (13 mg, 0.34 mmol), dry DMF (2.5 mL) and bromoacetonitrile (0.020 mL, 0.40 mmol). The mixture was stirred at 40 °C for 3 h. The residue was purified by column chromatography (silica gel, iso-hexane/ethyl acetate 3:2) to afford **AnnH79** as a white solid (92 mg, 80%). m.p. 208–209 °C; ^1^H-NMR (500 MHz, CDCl_3_) *δ* = 8.35 (d, *J* = 5.1 Hz, 1 H, 3-H), 7.95 (d, *J* = 8.3 Hz, 1 H, 5-H), 7.88 (d, *J* = 5.1 Hz, 1 H, 4-H), 7.53 (d, *J =* 8.3 Hz, 1 H, 6-H), 6.12 (s, 2 H, 1′-H); ^13^C-NMR (125 MHz, CDCl_3_) *δ* = 141.9, 138.5, 135.5, 135.3, 132.6, 124.9, 123.8, 123.3, 120.4, 116.9, 115.2, 114.3, 35.1; IR (KBr) $\tilde{\nu }$ (cm^−1^) = 3423, 3062, 2361, 1615, 1168; MS (ESI) *m/z =* 362, 360, 358, 356 [M^+^ + H], 238, 102; HRMS (ESI) calcd. for C_13_H_6_N_3_BrCl_2_ + H^+^: 353.9200; found 353.9194; purity: 100% (λ = 210 nm), 100% (λ = 254 nm) (by HPLC).

*1-Bromo-7,8-dichloro-9-(prop-2-yn-1-yl)-9H-pyrido[3,4-b]indole* (**AnnH80**). Prepared from **AnnnH52** (101 mg, 0.320 mmol), sodium hydride (17 mg, 0.43 mmol), dry DMF (2.5 mL) and propargyl bromide (80 wt % in toluene, 0.040 mL, 0.36 mmol). The mixture was stirred at 40 °C for 3 h. The residue was purified by column chromatography (silica gel, iso-hexane/ethyl acetate 3:2) to afford **AnnH80** as a beige solid (90 mg, 79%). m.p. 190–193 °C; ^1^H-NMR (500 MHz, CDCl_3_) *δ* = 8.30 (d, *J* = 5.0 Hz, 1 H, 3-H), 7.93 (d, *J* = 8.3, 1 H, 5-H), 7.87 (d, *J* = 5.0 Hz, 1 H, 4-H), 7.48 (d, *J* = 8.3 Hz, 1 H, 6-H), 6.07 (d, *J* = 2.4 Hz, 2 H, 1′-H), 2.33 (t, *J* = 2.4 Hz, 1 H, 3′-H); ^13^C-NMR (125 MHz, CDCl_3_) *δ* = 140.7, 138.8, 135.5, 135.0, 132.3, 124.0, 123.8, 123.1, 120.0, 117.0, 114.0, 79.0, 74.1, 36.1; IR (KBr) $\tilde{\nu }$ (cm^−1^) = 3433, 3293, 2125, 1614, 1053; MS (APCI) *m/z =* 361, 359, 357, 355 [M^+^+ H], 353, 279, 277, 275, 238, 102; HRMS (ESI) calcd. for C_14_H_7_N_2_BrCl_2_ + H^+^: 352.9247; found 352.9243; purity: 100% (λ = 210 nm), 100% (λ = 254 nm) (by HPLC).

*7-Methoxy-2,9-dihydro-1H-pyrido[3,4-b]indol-1-one* (**AnnH20**). To 7-methoxy-2,3,4,9-tetrahydro-1*H*-pyrido[3,4-*b*]indol-1-one (harmalacidine; **AnnH19**) [40] (440 mg, 2.03 mmol) in 20 mL of xylene was added *p*-chloranil (747 mg, 3.04 mmol). The mixture was stirred at reflux for 23 h, then cooled to room temperature and evaporated to dryness. The residue was purified by column chromatography (silica gel, iso-hexane/ethyl acetate/ethanol 2:2:1) to afford **AnnH20** as a beige solid (388 mg, 89%). ^1^H-NMR (500 MHz, DMSO-*d_6_*) *δ* = 11.80 (s, 1 H, NH), 11.31 (s, 1 H, NH), 7.88 (d, *J* = 8.7 Hz, 1 H, 5-H), 7.04 (d, *J* = 6.8 Hz, 1 H, 3-H), 6.93 (d, *J* = 2.2 Hz, 1 H, 8-H), 6.91 (d, *J* = 6.8 Hz, 1 H, 4-H), 6.80 (dd, *J* = 8.7, 2.3 Hz, 1-H, 6-H), 3.82 (s, 3 H, CH_3_); ^13^C-NMR (100 MHz, DMSO-*d_6_*) *δ =* 158.9, 155.3, 140.4, 127.3, 124.7 (2 C), 122.1, 116.0, 110.3, 99.4, 94.3, 55.2; IR (KBr) $\tilde{\nu }$ (cm^−1^) = 3430, 1639, 1560, 1262; MS (EI) *m/z* (relative intensity, %) = 214 (20) [M^+**•**^], 149 (18), 84 (40), 73 (100); HRMS (EI) calcd. for C_12_H_10_N_2_O_2_: 214.0742; found 214.0736; purity: 100% (λ = 210 nm), 100% (λ = 254 nm) (by HPLC).

*1-Chloro-7-methoxy-9-(prop-2-yn-1-yl)-9H-pyrido[3,4-b]indole* (**AnnH74**). Prepared from **AnnnH24** (61 mg, 0.26 mmol), sodium hydride (17 mg, 0.42 mmol), dry DMF (2.5 mL) and propargyl bromide (80 wt % in toluene, 0.030 mL, 0.26 mmol). The mixture was stirred at 40 °C for 4 h. The residue was purified by column chromatography (silica gel, iso-hexane/ethyl acetate 3:2) to afford **AnnH74** as a beige solid (53 mg, 74%). mp 141–144 °C; ^1^H-NMR (400 MHz, CDCl_3_) *δ* = 8.19 (d, *J* = 5.1 Hz, 1 H, 3-H), 7.97 (d, *J* = 8.4 Hz, 1 H, 5-H), 7.79 (d, *J* = 5.1 Hz, 1 H, 4-H), 7.00–6.97 (m, 2 H, 6-H, 8-H), 5.50 (d, *J* = 2.5 Hz, 2 H, 1′-H), 3.98 (s, 3 H, OCH_3_), 2.33 (t, *J* = 2.5 Hz, 1 H, 3′-H); ^13^C-NMR (125 MHz, CDCl_3_) *δ* = 161.7, 143.2, 139.0, 132.7, 132.6, 131.8, 122.6, 114.9, 113.5, 110.6, 93.6, 78.1, 73.2, 55.8, 34.2; IR (KBr) $\tilde{\nu }$ (cm^−1^) = 3428, 3159, 2108, 1627, 1168; MS (APCI) *m/z =* 273, 271 [M^+^+ H]; HRMS (ESI) calcd. for C_15_H_11_N_2_OCl + H^+^: 271.0638; found 271.0632; purity: 100% (λ = 210 nm), 99% (λ = 254 nm) (by HPLC).

*2-(1-Chloro-7-methoxy-9H-pyrido[3,4-b]indol-9-yl)acetonitrile* (**AnnH75**). Prepared from **AnnnH24** (96 mg, 0.41 mmol), sodium hydride (16 mg, 0.40 mmol), dry DMF (2.5 mL) and bromoacetonitrile (0.030 mL, 0.43 mmol). The mixture was stirred at 40 °C for 4 h. The residue was purified by column chromatography (silica gel, iso-hexane/ethyl acetate 3:2) to afford **AnnH75** as a beige solid (71 mg, 63%). m.p. 163–164 °C; ^1^H-NMR (400 MHz, CDCl_3_) *δ* = 8.25 (d, *J* = 5.1 Hz, 1 H, 3-H), 7.99 (d, *J* = 8.7 Hz, 1 H, 5-H), 7.81 (d, *J* = 5.1 Hz, 1 H, 4-H), 7.02 (dd, *J* = 8.1, 2.1 Hz, 1 H, 6-H), 6.92 (d, *J* = 2.1 Hz, 1 H, 8-H), 5.67 (s, 2 H, 1′-H), 3.99 (s, 3 H, OCH_3_); ^13^C-NMR (125 MHz, CDCl_3_) *δ* = 162.1, 142.7, 140.1, 133.3, 132.4, 131.5, 123.0, 115.1, 114.5, 113.8, 111.3, 93.3, 55.9, 32.9; IR (KBr) $\tilde{\nu }$ (cm^−1^) = 3434, 2926, 2363, 1624, 1204; MS (APCI) *m/z =* 274, 272 [M^+^+ H]; HRMS (ESI) calcd. for C_14_H_10_N_3_OCl + H^+^: 272.0590; found 272.0584; purity: 100% (λ = 210 nm), 99% (λ = 254 nm) (by HPLC).

*Ethyl 2-(1-Chloro-7-methoxy-9H-pyrido[3,4-b]indol-9-yl)acetate* (**AnnH76**). Prepared from **AnnnH24** (64 mg, 0.28 mmol), sodium hydride (20 mg, 0.50 mmol), dry DMF (2.5 mL) and ethyl bromoacetate (0.030 mL, 0.30 mmol). The mixture was stirred at 40 °C for 3 h. The residue was purified by column chromatography (silica gel, iso-hexane/ethyl acetate 3:2) to afford **AnnH76** as a beige solid (78 mg, 88%). m.p. 152–153 °C; ^1^H-NMR (500 MHz, CDCl_3_) *δ* = 8.18 (d, *J* = 5.1 Hz, 1 H, 3-H), 7.98 (d, *J* = 8.6, 1 H, 5-H), 7.80 (d, *J* = 5.1 Hz, 1 H, 4-H), 6.95 (dd, *J* = 8.6, 2.1 Hz, 1 H, 6-H), 6.78 (d, *J* = 2.1 Hz, 1 H, 8-H), 5.42 (s, 2 H, 1′-H), 4.25 (q, *J* = 7.1 Hz, 2 H, OCH_2_), 3.94 (s, 3 H, OCH_3_), 1.26 (t, *J* = 7.1 Hz, 3 H, CH_2_-CH_3_); ^13^C-NMR (125 MHz, CDCl_3_) *δ* = 168.6, 161.6, 143.7, 138.9, 132.6 (2 C), 122.8, 114.8 (2 C), 113.6, 110.3, 92.9, 61.9, 55.7, 46.1, 14.1; IR (KBr) $\tilde{\nu }$ (cm^−1^) = 3442, 3043, 1733, 1626, 1168; MS (APCI) *m/z =* 321, 319 [M^+^ + H]; HRMS (ESI) calcd. for C_16_H_15_N_2_O_3_Cl + H^+^: 319.0849; found 319.0844; purity: 98% (λ = 210 nm), 99% (λ = 254 nm) (by HPLC).

*3-(1-Chloro-7-methoxy-9H-pyrido[3,4-b]indol-9-yl)-N,N-dimethylpropan-1-amine* (**AnnH77**). Prepared from 1-chloro-7-methoxy-9*H*-pyrido[3,4-*b*]indole (**AnnH24**) (66 mg, 0.28 mmol), sodium hydride (41 mg, 1.0 mmol), dry THF (3 mL) and *N*,*N*-dimethyl-3-chloropropylamine hydrochloride (82 mg, 0.52 mmol). Beige solid (18 mg, 20%). m.p. 86–87 °C; ^1^H-NMR (500 MHz, CDCl_3_) *δ* = 8.15 (d, *J* = 5.1 Hz, 1 H, 3-H), 7.91 (d, *J* = 8.7 Hz, 1 H, 5-H), 7.78 (d, *J* = 5.1 Hz, 1 H, 4-H), 7.21 (d, *J* = 2.0 Hz, 1 H, 8-H), 6.91 (dd, *J* = 8.7, 2.1 Hz, 1 H, 6-H), 4.92 (t, *J* = 7.4 Hz, 2 H, 1′-H), 4.02 (s, 3 H, OCH_3_), 3.20–3.03 (m, 2 H, 3′-H), 2.80 (s, 6 H, N(CH_3_)_2_), 2.56–2.42 (m, 2 H, 2′-H); ^13^C-NMR (125 MHz, CDCl_3_) *δ* = 161.4, 143.7, 138.0, 132.4 (2 C), 132.2, 122.4, 114.5, 113.3, 110.0, 93.4, 56.4, 55.6, 45.5 (2 C), 42.3, 28.9; IR (KBr) $\tilde{\nu }$ (cm^−1^) = 3425, 2932, 1611, 1061; MS (APCI) *m/z =* 320, 318 [M^+^+ H], 282, 268; HRMS (ESI) calcd. for C_17_H_20_N_3_OCl + H^+^: 318.1373; found 318.1369; purity: 100% (λ = 210 nm), 100% (λ = 254 nm) (by HPLC).

*7-Methoxy-1-methyl-9H-carbazole***(AnnHOG3).** A solution of 100 mg (0.465 mmol) 7-methoxy-2,3,4,9-tetrahydro-1*H*-carbazol-1-one (**2**) [42] in 3 mL anhydrous THF under nitrogen was cooled to −35 °C and treated slowly under stirring with 0.620 mL of a 3M solution of methylmagnesium iodide (1.86 mmol). The solution was allowed to come to room temperature over 2 h and stirred for another 14 h, then quenched with 5 mL water and stirred for 30 min. The mixture was extracted with ethyl acetate (3 × 10 mL), the combined organic layers dried over sodium sulfate and evaporated. Purification by column chromatography (silica gel, iso-hexane/ethyl acetate 19:1) afforded the target compound as a pale brown solid (25 mg, 25%). m.p. 120 °C. ^1^H-NMR (DMSO-D_6_, 400 MHz): *δ* = 7.99 (s, 1 H, NH), 7.91 (d, J = 8.4 Hz, 1 H, 5-H), 7.82 (d, J = 6.4 Hz, 1 H, 3-H), 7.18 − 7.10 (m, 2 H, 2-H, 4-H), 6.97–6.95 (m, 1 H, 8-H), 6.86 (dd, J = 1.6 Hz, 8.8 Hz, 1 H, 6-H), 3.90 (s, 3 H, OCH_3_), 2.55 (s, 3 H, CH_3_). ^13^C-NMR (DMSO-D_6_, 125 MHz): *δ* = 158.92 (C-7), 140.72 (C-8a), 138.84 (C-1), 125.25 (C-2), 122.99 (C-4a), 121.17 (C-5), 119.65 (C-4), 119.44 (C-9a), 117.76 (C-4b), 117.13 (C-3), 108.12 (C-6), 94.83 (C-8), 55.60 (OCH_3_), 16.87 (CH_3_). IR (KBr): $\tilde{\nu }$ (cm^−1^) = 3439, 2925, 2854, 1628, 1610, 1500, 1448, 1280, 827, 780, 747. HR-MS (ESI -): *m/z* = 211.1003 (calcd. for für C_14_H_13_NO: 211.0997); purity: 96% (λ = 210 nm), 100% (λ = 254 nm) (by HPLC).

*1-Ethyl-7-methoxy-9H-pyrido[3,4-b]indole* (**AnnH89**). A dispersion of **AnnH24** (57 mg, 0.24 mmol), tetrakis(triphenylphosphine)palladium(0) (13.2 mg, 0.011 mmol) and potassium carbonate (68 mg) in 1.8 mL of dry DMF under a N_2_ atmosphere was treated with triethylborane (0.46 mL of a 1 M solution in hexane, 4.6 mmol) and stirred at reflux for 50 min. The mixture was worked up in the same manner as described for **AnnH88** to afford **AnnH89** as a beige solid (37 mg, 66%); as a by-product we obtained the dechlorination product norharmine (**AnnH90)** (9 mg, 18%) as a red–brown solid. Data for **AnnH89**: m.p. 109–117 °C; ^1^H-NMR (500 MHz, CDCl_3_) *δ* = 9.13 (s, NH), 8.36 (d, *J =* 5.3 Hz, 1 H, 3-H), 7.97 (d, *J* = 8.6 Hz, 1 H, 5-H), 7.73 (d, *J* = 5.3 Hz, 1 H, 4-H), 6.94 (d, *J* = 2.2 Hz, 1 H, 8-H), 6.89 (dd, *J* = 8.6, 2.2 Hz, 1 H, 6-H), 3.87 (s, 3 H, OCH_3_), 3.12 (q, *J* = 7.6 Hz, 2 H, 1′-H), 1.43 (t, *J* = 7.6 Hz, 3 H, 2′-H); ^13^C-NMR (125 MHz, CDCl_3_) *δ* = 161.0, 146.1, 141.8, 138.7, 134.0, 128.8, 122.5, 115.8, 112.2, 109.5, 94.7, 55.5, 27.2, 12.8; IR (KBr) $\tilde{\nu }$ (cm^−1^) = 3374, 3009, 1568, 1161; MS (ESI) *m/z =* 227 [M^+^+ H], 212; HRMS (ESI) calcd. for C_14_H_14_N_2_O+ H^+^: 227.1184; found 227.1177; purity: 61% (λ = 210 nm), 99% (λ = 210 nm) (by HPLC).

*Ethyl 2-(1-ethyl-7-methoxy-9H-pyrido[3,4-b]indol-9-yl)acetate* (**AnnH88**). A dispersion of **AnnH76** (49 mg, 0.15 mmol), tetrakis(triphenylphosphine)palladium(0) (7.9 mg, 0.0068 mmol) and potassium carbonate (42 mg) in 1.0 mL of dry DMF under a N_2_ atmosphere was treated with triethylborane (0.28 mL of 1 M solution in hexane, 2.8 mmol) and the mixture stirred at reflux for 2 h. The mixture was cooled to room temperature and water (10 mL) was added. The reaction mixture was extracted with ethyl acetate (4 × 10 mL), the combined organic layers were dried over magnesium sulfate and evaporated to dryness. The residue was purified by column chromatography (silica gel, iso-hexane/ethyl acetate/ethanol 2:2:1) and by precipitation with using of DCM and *n*-hexane to afford **AnnH88** as a beige solid (33 mg, 69%). m.p. 95–97 °C; ^1^H-NMR (500 MHz, CDCl_3_) *δ* = 8.35 (d, *J =* 5.1 Hz, 1 H, 3-H), 7.96 (d, *J* = 8.6 Hz, 1 H, 5-H), 7.72 (d, *J* = 5.1 Hz, 1 H, 4-H), 6.89 (dd, *J* = 8.6, 2.1 Hz, 1 H, 6-H), 6.75 (d, *J* = 2.1 Hz, 1 H, 8-H), 5.15 (s, 2 H, 1′-H), 4.22 (q, *J* = 7.1 Hz, 2 H, O-CH_2_), 3.91 (s, 3 H, OCH_3_), 3.17 (q, *J* = 7.6 Hz, 2 H, 1′′-H), 1.41 (t, *J* = 7.6 Hz, 3 H, 2′′-H), 1.22 (t, *J* = 7.1 Hz, 3 H, OCH_2_-CH_3_); ^13^C-NMR (125 MHz, CDCl_3_) *δ* = 168.7, 161.1, 145.7, 143.5, 139.2, 134.9, 130.2, 122.4, 115.6, 112.2, 109.3, 93.2, 61.9, 55.7, 47.0, 28.6, 14.1, 14.0; IR (KBr) $\tilde{\nu }$ (cm^−1^) = 3423, 2966, 1747, 1624, 1431, 1167; MS (ESI) *m/z =* 313 [M^+^+ H], 279; HRMS (ESI) calcd. for C_18_H_20_N_2_O_3_+ H^+^: 313.1552; found 313.1548; purity: 80% (λ = 210 nm), 100% (λ = 254 nm) (by HPLC).

*Ethyl 4-(4-methoxyphenyl)-2-methylpyridine-3-carboxylate* (**3**). A solution of *(E)*-4-methoxycinnamaldehyde (3.2 g, 0.019 mol), ethyl 3-aminocrotonate (2.1 mL, 0.017 mol) and 0.080 mL piperidine in 20 mL ethanol was refluxed for 1 h, then evaporated to dryness. The residue was suspended in 70 mL ethanol and treated with 0.34 g (4.0 mmol) sodium bicarbonate. Then, a 10% ethanolic solution of iodine was added dropwise under stirring until the colour change from yellow to brown persisted for >2 min. Then, 0.1 M sodium thiosulfate solution was added until the colour changed back to yellow. The mixture was evaporated to dryness and the residue taken up in 20 mL diethyl ether. The solution was washed with 20 mL water and 20 mL brine, dried over magnesium sulfate and evaporated. The oily residue is a mixture of **3** and its less polar 6-aryl isomer. Purification by column chromatography (silica gel, iso-hexane/ethyl acetate 65:35) afforded the target compound as a brown oil. Yield: 800 mg (16%). ^1^H-NMR (CDCl_3_, 500 MHz): *δ* = 8.53 (d, *J* = 5.2 Hz, 1 H, 6-H), 7.38–7.29 (m, 2 H, 2′/6′-, 3′/5′-H), 7.14 (d, *J* = 5.2 Hz, 1 H, 5-H), 7.01–6.89 (m, 2 H, 2′/6′-, 3′/5′-H), 4.17 (q, *J* = 7.1 Hz, 2 H, CH_2_), 3.85 (s, 3 H, OCH_3_), 2.62 (s, 3 H, Ar-CH_3_), 1.09 (t, *J* = 7.1. Hz, 3 H, CH_2_-CH_3_); ^13^C-NMR (CDCl_3_, 100 MHz): *δ* = 168.9 (C=O), 160.0 (C-4′), 155.4 (C-2), 149.5 (C-6), 147.5 (C-4), 130.6 (C-1′), 129.2 (C-2′/6′, -3′/5′), 128.5 (C-3), 121.6 (C-5,6), 114.0 (C-2′/6′, C-3′/5′), 61.4 (CH_2_), 55.3 (OCH_3_), 22.8 (Ar-CH_3_), 13.8 (CH_3_); MS (APCI): *m/z =* 272 [M^+^+ H], 102; IR (KBr): $\tilde{\nu }$ (cm^−1^) = 3401, 3038, 2979, 2960, 2838, 1726, 1610, 1579, 1548, 1515, 1463, 1443, 1401, 1366, 1294, 1251, 1215, 1180, 1140, 1113, 1097, 1073, 1032, 853, 829; HR-MS: *m/z* = 271.1207 (calcd. for C_16_H_17_NO_3_: 271.1208); purity: 100% (λ = 210 nm), 100% (λ = 254 nm) (by HPLC).

*7-Methoxy-1-methyl-9H-indeno-[2,1-c]pyridin-9-one* (**AnnHT2**). A mixture of 387 mg (1.43 mmol) ester 3 and 17 g polyphosphoric acid was stirred at 135 °C for 5 h. After cooling to room temperature, the brown viscous mixture was taken up in 80 mL ice-water and stirred for 30 min. The mixture was adjusted to pH 10 with 6N NaOH and extracted with ethyl acetate (4 × 200 mL). The combined organic layers were dried over magnesium sulfate and evaporated. Purification by column chromatography (silica gel, iso-hexane/ethyl acetate/ethanol 2:2:1) afforded the target compound as a yellow solid (45 mg, 14%). m.p. 140–143 °C. ^1^H-NMR (DMSO-D_6_, 400 MHz): *δ* = 8.55 (d, *J* = 4.9 Hz, 1 H, 3-H), 7.80 (d, *J* = 8.2 Hz, 1 H, 5-H), 7.57 (d, *J* = 4.9 Hz, 1 H, 4-H), 7.19 (dd, *J* = 8.2, 2.5 Hz, 1 H, 6-H), 7.15 (d, *J* = 2.4 Hz, 1 H, 8-H), 3.86 (s, 3 H, OCH_3_), 2.64 (s, 3 H, CH_3_); ^13^C-NMR (DMSO-D_6_, 125 MHz): *δ* = 193.3 (C=O), 162.2 (C-7), 156.1 (C-1), 155.4 (C-3), 152.2 (C-4a), 135.2 (C-8a), 133.2 (C-4b), 124.5 (C-9a), 123.9 (C-5), 120.3 (C-6/8) 113.6 (C-4), 109.1 (C-6/8), 55.9 (OCH_3_), 20.5 (CH_3_); MS (CI): *m/z* (rel. int. in %) = 226 [M^+^ + H] (28), 125 (10), 97 (12), 79 (100); MS (EI): *m/z* (rel. Int. in %) = 225 [M^+•^] (100), 210 (30), 182 (18), 154 (26), 84 (30); IR (KBr): $\tilde{\nu }$ (cm^−1^) = 3442, 2924, 2854, 2360, 2342, 1708, 1636, 1618, 1593, 1484, 1453, 1438, 1369, 1343, 1290, 1261, 1240, 1213, 1195, 1149, 1061, 1019, 950, 881, 827, 794, 681; HR-MS (EI): *m/z* = 225.0800 (calcd. for C_14_H_11_NO_2_: 225.0790); purity: 93% (λ = 210 nm), 96% (λ = 254 nm) (by HPLC).

*7-Hydroxy-1-methyl-9H-indeno-[2,1-c]pyridin-9-one* (**AnnHT6**). A suspension of 100 mg (0.369 mmol) of ester 3 in 1.5 mL trifluoromethanesulfonic acid was stirred under nitrogen for 4 h at 85 °C, then cooled to room temperature and neutralized carefully with ice-cooled saturated aqueous sodium carbonate solution. The mixture was extracted with ethyl acetate (3 × 50 mL), the combined organic layers were washed with brine (2 × 50 mL), dried over magnesium sulfate and evaporated. Purification by column chromatography (silica gel, iso-hexane/ethyl acetate 65:35) afforded the target compound as a yellow solid (36 mg, 46%). m.p. 275–276 °C. ^1^H-NMR (DMSO-D_6_, 400 MHz): *δ* (ppm) = 10.40 (s, 1 H, OH), 8.53 (d, *J* = 4.9 Hz, 1 H, 3-H), 7.72 (d, *J* = 8.7 Hz, 1 H, 5-H), 7.54 (d, *J* = 4.5 Hz, 1 H, 4-H), 7.03–6.99 (m, 2 H, 6-H, 8-H), 2.64 (s, 1 H, CH_3_); ^13^C-NMR (DMSO-D_6_, 125 MHz): *δ* (ppm) = 193.7 (C=O), 160.7 (C-7), 156.0 (C-1), 153.4 (C-3), 152.7 (C-4a), 135.5 (C-8a/4b), 131.6 (C-8a/4b), 124.5 (C-9a), 124.1 (C-5), 121.1 (C-6), 113.3 (C-4), 110.7 (C-8), 20.5 (CH_3_); MS (APCI): *m/z* (rel. int. in %) = 212 [M^+^+ H], 186, 102; IR (KBr): $\tilde{\nu }$ (cm^−1^) = 3443, 2924, 2854, 1716, 1592, 1456, 1380, 1298, 1248, 1231, 1209, 1144, 1059, 965, 849; HR-MS (ESI): *m/z* = 212.0709 (calcd. for C_13_H_10_NO_2_: 212.0712); purity: 90% (λ = 210 nm), 100% (λ = 254 nm) (by HPLC).

*(±)-7-Methoxy-1-methyl-9H-indeno-[2,1-c]pyridin-9-ol* (**AnnHT3**). An ice-cooled suspension of 96 mg (0.42 mmol) ketone AnnHT2 in 7 mL methanol was treated with 23 mg (0.61 mmol) sodium borohydride and then stirred for 15 h at room temperature. The methanol was removed by evaporation and the residue treated with 13 mL dichloromethane and 7 mL aqueous 15% potassium hydroxide solution and stirred for 1 h. The organic layer was separated, washed with water (2 × 10 mL), dried over magnesium sulfate and evaporated. Purification by column chromatography (silica gel, iso-hexane/ethyl acetate/ethanol 2:2:1) afforded the target compound as a yellow solid (47 mg, 49%). m.p. 191–192 °C. ^1^H-NMR (DMSO-D_6_, 400 MHz): *δ* = 8.38 (d, *J* = 5.0 Hz, 1 H, 3-H), 7.78 (d, *J* = 8.3 Hz, 1 H, 5-H), 7.52 (d, *J* = 5.0 Hz, 1 H, 4-H), 7.21 (d, *J* = 2.4 Hz, 1 H, 8-H), 7.00 (dd, *J* = 8.3, 2.4 Hz, 1 H, 6-H), 5.93 (s, 1 H, OH), 3.84 (s, 3 H, OCH_3_), 2.58 (s, 3 H, CH_3_); ^13^C-NMR (DMSO-D_6_, 125 MHz): *δ* = 161.5 (C-7), 155.3 (C-1), 150.5 (C-8a), 149.6 (C-3), 147.8 (C-4a), 138.3 (C-9a), 130.3 (C-4b), 123.1 (C-5), 115.1 (C-6), 112.8 (C-4), 111.2 (C-8), 73.4 (C-9), 55.9 (OCH_3_), 21.6 (CH_3_); MS (APCI): *m/z* (rel. int. in %) = 229 [M^+^+ H], 213, 102; IR (KBr): $\tilde{\nu }$ (cm^−1^) = 3423, 2921, 2836, 2599, 2361, 2343, 1708, 1592, 1483, 1438, 1369, 1342, 1290, 1239, 1212, 1148, 1103, 1060, 1019, 949, 826, 794; HR-MS (EI): *m/z* = 227.0953 (calcd for C_14_H_13_NO_2_: 227.0946); purity: 100% (λ = 210 nm), 100% (λ = 254 nm) (by HPLC).

*(2E)-(7-Methoxy-1-methyl-9H-indeno[2,1c]pyridin-9-ylidene)ethanenitrile* (**AnnHT5**). A suspension of 20 mg (0.50 mmol) sodium hydride (60% in mineral oil) in 1.25 mL THF was treated with 0.040 mL (0.30 mmol) diethyl cyanomethylphosphonate and stirred at room temperature for 15 min. Then, a solution of 56 mg (0.25 mmol) ketone AnnHT2 in 1.25 mL THF was added slowly with stirring. The mixture was stirred for another 17 h and then evaporated to dryness. The residue was taken up in 20 mL dichloromethane, the solution washed with 25 mL water, dried over magnesium sulfate, and evaporated. Purification by column chromatography (silica gel, iso-hexane/ethyl acetate/ethanol 2:2:1) afforded a yellow solid. This product was disssolved in dichloromethane and precipitated with n-hexane to give the target compound as a yellow solid (13 mg, 22%). ^1^H-NMR (DMSO-D_6_, 400 MHz): *δ* = 8.47 (d, *J* = 5.0 Hz, 1 H, 3-H), 8.08 (s, 1 H, 8-H), 7.60 (d, *J* = 8.4 Hz, 1 H, 5-H), 7.34 (d, *J* = 5.0 Hz, 1 H, 4-H), 7.04 (d, *J* = 8.4 Hz, 1 H, 6-H), 6.28 (s, 1 H, 1′-H), 3.92 (s, 3 H, OCH_3_), 2.76 (s, 1 H, CH_3_); ^13^C-NMR (DMSO-D_6_, 125 MHz): *δ* = 162.0 (C-7), 154.4 (C-1), 153.1 (CN), 151.1 (C-3), 148.8 (C-4a), 137.5 (C-8a), 131.7 (C-4b), 129.0 (C-9a), 122.4 (C-5), 118.2 (C-6), 117.1 (C-9), 112.7 (C-4), 110.2 (C-8), 93.3 (C-1′), 55.7 (OCH_3_), 24.5 (CH_3_); MS (APCI): *m/z* (rel. int. in %) = 249 [M^+^+ H], 102; IR (KBr): $\tilde{\nu }$ (cm^−1^) = 3443, 3088, 2987, 2966, 2838, 225, 2204, 1619, 1585, 1484, 1458, 1141, 1403, 1371, 1340, 1325, 1283, 1259, 1242, 1139, 1063, 1027, 974, 856, 829, 814; HR-MS (ESI): *m/z* = 249.1025 (calcd. for C_16_H_13_N_2_O: 249.1028); purity: 100% (λ = 210 nm), 100% (λ = 254 nm) (by HPLC).

## Figures and Tables

**Table 1:** Results of in vitro screening of the harmine analogues on MAO-A, DYRK1A and CLK1 ^1^.

Compound	MAO-A	DYRK1A	CLK1
Residual activities in % at 1 µM inhibitor concentration			
*Alkaloids and direct modification at ring A*			
harmine	3	27	23
harmane	18	97	-
norharmine (**AnnH90**)	45	36	26 ***
harmol	120	18	6 ***
**AnnH18**	100	27	58
*Direct modifications at ring C*			
**AnnH6_1**	16	97	-
**AnnH6_3_2**	9	92	-
**AnnH5_2**	7	88	-
**AnnH5_3**	8	95	-
**AnnH6_3_1**	64	99	-
**AnnH5_1**	70	101	-
**AnnH5_4**	-	126	-
**AnnH9**	8	96	-
**AnnH11**	4	104	60 ***
**AnnH14**	56	94	62 ***
**AnnH62**	22	95	100 ***
*Modifications at ring B—N-acylation and N–alkylation at N-9*			
**AnnH59**	19	30	15 ***
**AnnH1**	2	24	6
**AnnH3**	10	26	24
**AnnH44**	22	2	17
**AnnH70**	18	6	17 ***
**AnnH73**	52	6	2 ***
**AnnH4**	80	88	-
**AnnH35**	128	100	80 ***
**AnnH38**	127	12	31
**AnnH39**	125	91	84
**AnnH22**	39	3	15 ***
**AnnH40**	30	40	37
**AnnH36**	19	59	62
**AnnH16**	103	107	-
**AnnH21**	75	15	70
**AnnH53**	70	33	77
**AnnH55**	67	48	85
**AnnH57**	97	33	78
**AnnH12**	101	30	45
**AnnH26**	59	2	42 ***
**AnnH27**	72	4	79
**AnnH32**	105	12	44
**AnnH25**	6	40	34 ***
**AnnH28**	131	36	89
**AnnH30**	24	64	87
**AnnH34**	130	75	78 ***
**AnnH31**	75	8	11
**AnnH42**	114	22	44
**AnnH43**	102	5	10
*De novo-synthesis: harmine-bauerine C hybrids*			
**AnnH65**	-	100 *	-
**AnnH52**	118	36	45
**AnnH63**	32	3	26
**AnnH66**	78	26	71 ***
**AnnH69**	101	17	33
**AnnH67**	122	17	34
**AnnH71**	110	47	90 ***
**AnnH79**	143	93	88
**AnnH80**	123	95	84
*Elinination/reduction of the basicity at N-2*			
**AnnH19**	--	122	>90
**AnnH20**	127	121	-
**AnnH24**	103	10	17
**AnnH74**	112	12	46
**AnnH75**	119	10	24
**AnnH76**	112	35	96
**AnnH77**	123	63	86 ***
**AnnHOG3**	100	71	97
*1-Ethyl analogues of harmine*			
**AnnH89**	34	37	7 ***
**AnnH88**	137	47	52 ***
*Replacement of NH in ring C by ketone and carbinol*			
**AnnHT2**	15	55	54
**AnnHT6**	46	37	27
**AnnHT3**	34	59	76
**AnnHT5**	65 **	35	7

^1^ Values are mean values of at least three (MAO-A and DYRK1A) or at least two independent experiments (CLK1). Values >100% can be due to experimental variability or inhibition of luciferase activity in the Kinase-Glo^®^ assay. “-“: not tested. * Value not reliable due to very poor solubility, ** value not reliable due to fluctuating individual values. *** Experiments performed in duplicate.

**Table 2:** Inhibition of MAO-A and DYRK1A by selected β-carbolines (95% confidence intervals in parentheses).

Compound	MAO-A IC_50_ (nM)	DYRK1A IC_50_ (nM)	Selectivity for DYRK1A over MAO-A
harmine	107	33–80	<3
**AnnH31**	3240 (2012–5202)	81 (53.6–122)	40
**AnnH43**	5390 (3108–9346)	202 (186–218)	26
**AnnH75**	**>10,000**	181 (140–234)	**>55**

**Table 3:** Crystal structure data of the DYRK1A-**AnnH75** complex (PDB 4YU2)

Complex	DYRK1A-AnnH75
PDB Accession Code	4YU2
*Data Collection*	
Beamline	Diamond, beamline I04-1
Wavelength (Å)	0.9200
Resolution ^a^ (Å)	48.34–2.90 (3.06–2.90)
Spacegroup	*C* 2
Cell dimensions	*a* = 265.6, *b* = 65.4, *c =* 138.7 Å
	α = γ = 90.0°, β = 114.4°
No. unique reflections ^a^	48,349 (6999)
Completeness ^a^ (%)	99.5 (99.5)
I/σI ^a^	10.6 (2.0)
R_merge_^a^	0.126 (0.928)
Redundancy ^a^	6.7 (6.8)
*Refinement*	
ligands	AnnH75
No. atoms in refinement (P/L/O) ^b^	11,321/76/365
R_fact_ (%)	24.3
R_free_ (%)	27.7
B_f_ (P/L/O) ^b^ (Å^2^)	76/78/50
rms deviation bond ^c^ (Å)	0.011
rms deviation angle ^c^ (°)	1.2
*Molprobity*	
Ramachandran favour	92.8
Ramachandran allowed	99.9

^a^ Values in brackets show the statistics for the highest resolution shells. ^b^ P/L/O indicate protein, ligand molecule, and other (water and solvent molecules), respectively. ^c^ rms indicates root-mean-square.

## References

[1] Becker W., Sippl W. (2011). Activation, regulation, and inhibition of DYRK1A. FEBS J..

[2] Jarhad D.B., Mashelkar K.K., Kim H.-R., Noh M., Jeong L.S. (2018). Dual-Specificity Tyrosine Phosphorylation-Regulated Kinase 1A (DYRK1A) Inhibitors as Potential Therapeutics. J. Med. Chem..

[3] Becker W., Soppa U., Tejedor F.J. (2014). DYRK1A: A potential drug target for multiple Down syndrome neuropathologies. CNS Neurol. Disord. Drug Targets.

[4] Wegiel J., Gong C.-X., Hwang Y.-W. (2011). The role of DYRK1A in neurodegenerative diseases. FEBS J..

[5] Boni J., Rubio-Perez C., López-Bigas N., Fillat C., De la Luna S. (2020). The DYRK Family of Kinases in Cancer: Molecular Functions and Therapeutic Opportunities. Cancers.

[6] Belgardt B.F., Lammert E. (2016). DYRK1A: A Promising Drug Target for Islet Transplant-Based Diabetes Therapies. Diabetes.

[7] Ackeifi C., Swartz E., Kumar K., Liu H., Chalada S., Karakose E., Scott D.K., Garcia-Ocaña A., Sanchez R., DeVita R.J. (2020). Pharmacologic and genetic approaches define human pancreatic β cell mitogenic targets of DYRK1A inhibitors. JCI Insight.

[8] Kumar K., Man-Un Ung P., Wang P., Wang H., Li H., Andrews M.K., Stewart A.F., Schlessinger A., DeVita R.J. (2018). Novel selective thiadiazine DYRK1A inhibitor lead scaffold with human pancreatic β-cell proliferation activity. Eur. J. Med. Chem..

[9] Darwish S.S., Abdel-Halim M., Salah M., Abadi A.H., Becker W., Engel M. (2018). Development of novel 2,4-bispyridyl thiophene-based compounds as highly potent and selective Dyrk1A inhibitors. Part I: Benzamide and benzylamide derivatives. Eur. J. Med. Chem..

[10] Tazarki H., Zeinyeh W., Esvan Y.J., Knapp S., Chatterjee D., Schröder M., Joerger A.C., Khiari J., Josselin B., Baratte B. (2019). New pyrido [3,4-g]quinazoline derivatives as CLK1 and DYRK1A inhibitors: Synthesis, biological evaluation and binding mode analysis. Eur. J. Med. Chem..

[11] Lechner C., Flaßhoff M., Falke H., Preu L., Loaëc N., Meijer L., Knapp S., Chaikuad A., Kunick C. (2019). [b]-Annulated Halogen-Substituted Indoles as Potential DYRK1A Inhibitors. Molecules.

[12] Bain J., Plater L., Elliott M., Shpiro N., Hastie C.J., McLauchlan H., Klevernic I., Arthur J.S.C., Alessi D.R., Cohen P. (2007). The selectivity of protein kinase inhibitors: A further update. Biochem. J..

[13] Tahtouh T., Elkins J.M., Filippakopoulos P., Soundararajan M., Burgy G., Durieu E., Cochet C., Schmid R.S., Lo D.C., Delhommel F. (2012). Selectivity, Cocrystal Structures, and Neuroprotective Properties of Leucettines, a Family of Protein Kinase Inhibitors Derived from the Marine Sponge Alkaloid Leucettamine B. J. Med. Chem..

[14] Göckler N., Jofre G., Papadopoulos C., Soppa U., Tejedor F.J., Becker W. (2009). Harmine specifically inhibits protein kinase DYRK1A and interferes with neurite formation. FEBS J..

[15] Kim H., Sablin S.O., Ramsay R.R. (1997). Inhibition of monoamine oxidase A by beta-carboline derivatives. Arch. Biochem. Biophys..

[16] Ogawa Y., Nonaka Y., Goto T., Ohnishi E., Hiramatsu T., Kii I., Yoshida M., Ikura T., Onogi H., Shibuya H. (2010). Development of a novel selective inhibitor of the Down syndrome-related kinase Dyrk1A. Nat. Commun..

[17] Son S.Y., Ma J., Kondou Y., Yoshimura M., Yamashita E., Tsukihara T. (2008). Structure of human monoamine oxidase A at 2.2-A resolution: The control of opening the entry for substrates/inhibitors. Proc. Natl. Acad. Sci. USA.

[18] Reniers J., Robert S., Frederick R., Masereel B., Vincent S., Wouters J. (2011). Synthesis and evaluation of β-carboline derivatives as potential monoamine oxidase inhibitors. Bioorg. Med. Chem..

[19] Haider S., Alhusban M., Chaurasiya N.D., Tekwani B.L., Chittiboyina A.G., Khan I.A. (2018). Isoform selectivity of harmine-conjugated 1,2,3-triazoles against human monoamine oxidase. Fut. Med. Chem..

[20] Drung B., Scholz C., Barbosa V.A., Nazari A., Sarragiotto M.H., Schmidt B. (2014). Computational & experimental evaluation of the structure/activity relationship of β-carbolines as DYRK1A inhibitors. Bioorg. Med. Chem. Lett..

[21] Bálint B., Wéber C., Cruzalegui F., Burbridge M., Kotschy A. (2017). Structure-Based Design and Synthesis of Harmine Derivatives with Different Selectivity Profiles in Kinase versus Monoamine Oxidase Inhibition. ChemMedChem.

[22] Rüben K., Wurzlbauer A., Walte A., Sippl W., Bracher F., Becker W. (2015). Selectivity Profiling and Biological Activity of Novel β-Carbolines as Potent and Selective DYRK1 Kinase Inhibitors. PLoS ONE.

[23] Kumar K., Wang P., Sanchez R., Swartz E.A., Stewart A.F., DeVita R.J. (2018). Development of Kinase-Selective, Harmine-Based DYRK1A Inhibitors that Induce Pancreatic Human β-Cell Proliferation. J. Med. Chem..

[24] Frost D., Meechoovet B., Wang T., Gately S., Giorgetti M., Shcherbakova I., Dunckley T. (2011). β-Carboline Compounds, Including Harmine, Inhibit DYRK1A and Tau Phosphorylation at Multiple Alzheimer’s Disease-Related Sites. PLoS ONE.

[25] Fedorov O., Huber K., Eisenreich A., Filippakopoulos P., King O., Bullock A.N., Szklarczyk D., Jensen L.J., Fabbro D., Trappe J. (2011). Specific CLK inhibitors from a novel chemotype for regulation of alternative splicing. Chem. Biol..

[26] Huber K., Brault L., Fedorov O., Gasser C., Filippakopoulos P., Bullock A.N., Fabbro D., Trappe J., Schwaller J., Knapp S. (2012). 7,8-Dichloro-1-oxo-β-carbolines as a Versatile Scaffold for the Development of Potent and Selective Kinase Inhibitors with Unusual Binding Modes. J. Med. Chem..

[27] Schröder M., Bullock A.N., Fedorov O., Bracher F., Chaikuad A., Knapp S. (2020). DFG-1 Residue Controls Inhibitor Binding Mode and Affinity, Providing a Basis for Rational Design of Kinase Inhibitor Selectivity. J. Med. Chem..

[28] Li H.-Y., Koike K., Ohmoto T. (1993). New Alkaloids, Picrasidines W, X and Y, from Picrasma quassioides and X-Ray Crystallographic Analysis of Picrasidine Q. Chem. Pharm. Bull..

[29] Ponce M.A., Tarzi O.I., Erra-Balsells R. (2003). Synthesis and isolation of chloro-β-carbolines obtained by chlorination of β-carboline alkaloids in solution and in solid state. J. Heterocycl. Chem..

[30] Ponce M.A., Erra-Balsells R. (2001). Synthesis and isolation of bromo-β-carbolines obtained by bromination of β-carboline alkaloids. J. Heterocycl. Chem..

[31] Cao R., Yi W., Wu Q., Guan X., Feng M., Ma C., Chen Z., Song H., Peng W. (2008). Synthesis and cytotoxic activities of 1-benzylidine substituted β-carboline derivatives. Bioorg. Med. Chem. Lett..

[32] Dunckley T. (2012). Compounds that inhibit tau phosphorylation. Patent WO.

[33] Cuny G.D., Ulyanova N.P., Patnaik D., Liu J.F., Lin X., Auerbach K., Ray S.S., Xian J., Glicksman M.A., Stein R.L. (2012). Structure-activity relationship study of beta-carboline derivatives as haspin kinase inhibitors. Bioorg. Med. Chem. Lett..

[34] Cao R., Chen Q., Hou X., Chen H., Guan H., Ma Y., Peng W., Xu A. (2004). Synthesis, acute toxicities, and antitumor effects of novel 9-substituted beta-carboline derivatives. Bioorg. Med. Chem..

[35] Bethe B., Junge B., Lieb F., Nielsch U., Sperzel M., Velten R. (1999). Verwendung von ß-Carbolinderivaten zur Bekämpfung von TNF-a-abhängigen Krankheiten. Patent DE.

[36] Pohl B., Luchterhandt T., Bracher F. (2007). Total Syntheses of the Chlorinated β-Carboline Alkaloids Bauerine A, B, and C. Syn. Commun..

[37] Bruel A., Bénéteau R., Chabanne M., Lozach O., Le Guevel R., Ravache M., Bénédetti H., Meijer L., Logé C., Robert J.-M. (2014). Synthesis of new pyridazino [4,5-b]indol-4-ones and pyridazin-3(2H)-one analogs as DYRK1A inhibitors. Bioorg. Med. Chem. Lett..

[38] Bracher F., Hildebrand D. (1994). 1,9-Dimetalated ß-carbolines. Versatile building blocks for the total synthesis of Alkaloids. Tetrahedron.

[39] Bracher F., Hildebrand D. (1993). ß-Carbolin-Alkaloide, IV.-Synthese von 1-Alkyl-ß-carbolinen und Strukturrevision von Lycii-Alkaloid I. Liebigs Ann. Chem..

[40] Bracher F., Hildebrand D. (1993). ß-Carbolin-Alkaloide, 3. Mitt.: Synthese von Harmalacidin und Strychnocarpin. Pharmazie.

[41] Bracher F., Hildebrand D. (1992). ß-Carbolin-Alkaloide, I. Synthese von 1-Aryl- und 1-Alkenyl-ß-carbolinen durch Palladium-katalysierte Kupplungsreaktionen. Liebigs Ann. Chem..

[42] Douglas B., Kirkpatrick J.L., Moore B.P., Weisbach J.A. (1964). Alkaloids of *Ochrosia poweri* Bailey. II. The 2-acylindole stem-bark bases. Austr. J. Chem..

[43] Weller D.D., Rapoport H. (1976). Synthesis of cis- and trans-4a-Phenyldecahydroisoquinolines. J. Am. Chem. Soc..

[44] Bracher F. (1989). Polycyclische aromatische Alkaloide, 2.Mitt. Synthese von Onychin und Eupolauridin. Arch. Pharm..

[45] Soundararajan M., Roos A.K., Savitsky P., Filippakopoulos P., Kettenbach A.N., Olsen J.V., Gerber S.A., Eswaran J., Knapp S., Elkins J.M. (2013). Structures of Down syndrome kinases, DYRKs, reveal mechanisms of kinase activation and substrate recognition. Structure.

[46] Kabsch W. (2010). XDS. Acta Crystallogr. D Biol. Crystallogr..

[47] (1994). Collaborative Computational Project, Number 4. The CCP4 suite: Programs for protein crystallography. Acta Crystallogr. D Biol. Crystallogr..

[48] McCoy A.J., Grosse-Kunstleve R.W., Adams P.D., Winn M.D., Storoni L.C., Read R.J. (2007). Phaser crystallographic software. J. Appl. Crystallogr..

[49] Emsley P., Lohkamp B., Scott W.G., Cowtan K. (2010). Features and development of Coot. Acta Crystallogr. D Biol. Crystallogr..

[50] Murshudov G.N., Vagin A.A., Dodson E.J. (1997). Refinement of Macromolecular Structures by the Maximum-Likelihood Method. Acta Crystallogr. D Biol. Crystallogr..

[51] Painter J., Merritt E.A. (2006). Optimal description of a protein structure in terms of multiple groups undergoing TLS motion. Acta Crystallogr. D Biol. Crystallogr..

[52] Davis I.W., Leaver-Fay A., Chen V.B., Block J.N., Kapral G.J., Wang X., Murray L.W., Arendall W.B., Snoeyink J., Richardson J.S. (2007). MolProbity: All-atom contacts and structure validation for proteins and nucleic acids. Nucleic Acids Res..

[53] Untergehrer M., Bracher F. (2020). A short divergent approach to highly substituted carbazoles and β-carbolines via in situ-generated diketoindoles. Tetrahedron Lett..

[54] Kamlah A., Bracher F. (2020). A novel approach to highly substituted β-carbolines via reductive ring transformation of 2-acyl-3-isoxazolylindoles. Eur. J. Org. Chem..

